# Diagnostic and therapeutic innovations in myocardial infarction: a focus on mitochondrial dysfunction

**DOI:** 10.3389/fcvm.2026.1748411

**Published:** 2026-05-08

**Authors:** Xingli Gao, Yajie Pan, Qian Liu, Hailang Wu

**Affiliations:** 1 Department of Cardiology, Union Hospital, Tongji Medical College, Huazhong University of Science and Technology, Wuhan, China; 2 Hubei Key Laboratory of Biological Targeted Therapy, Union Hospital, Tongji Medical College, Huazhong University of Science and Technology, Wuhan, China; 3 Hubei Provincial Engineering Research Center of Immunological Diagnosis and Therapy for Cardiovascular Diseases, Union Hospital, Tongji Medical College, Huazhong University of Science and Technology, Wuhan, China; 4 Key Laboratory of Biological Targeted Therapy (Huazhong University of Science and Technology), Ministry of Education, Wuhan, China; 5 Center for Reproductive Medicine, Wuhan Children’s Hospital, Tongji Medical College, Huazhong University of Science and Technology, Wuhan, China

**Keywords:** biomarkers, mitochondrial dysfunction, myocardial infarction, reactive oxygen species, therapeutic strategy

## Abstract

Myocardial infarction (MI), a critical condition within the spectrum of cardiovascular diseases, is a leading cause of mortality and morbidity globally. This review focuses on the role of mitochondrial dysfunction in MI, covering fundamental theories, epidemiology, diagnostic approaches, therapeutic strategies, current debates, and future research directions. It explores the development of concepts, underlying pathophysiological mechanisms, and the impact of mitochondrial genetics on MI. The review addresses epidemiological factors, including prevalence, demographic disparities, and associated risk factors. Furthermore, it provides a comprehensive analysis of diagnostic methodologies, encompassing biomarkers, imaging techniques, and molecular diagnostics. The discourse expands to include therapeutic strategies, incorporating pharmacological interventions, gene therapy, and lifestyle modifications. It provides a critical analysis of the controversies regarding the causative role of mitochondrial dysfunction, the challenges in translating research findings into clinical practice, and the associated ethical considerations. Moreover, the review delves into emerging technologies, the potential for personalized medicine, and the long-term prognosis of MI survivors, with the objective of offering a comprehensive understanding of this complex field to inform future research and clinical applications.

## Graphical Abstract

**Figure:**
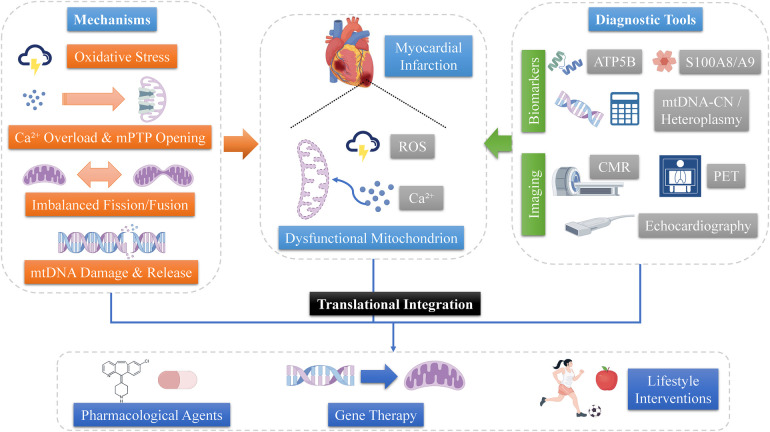

This graphical abstract illustrates the central role of mitochondrial dysfunction in myocardial infarction (MI). The left panel summarizes key pathophysiological mechanisms (oxidative stress, calcium overload, mitochondrial dynamics imbalance, and mtDNA damage). The right panel highlights diagnostic biomarkers (ATP5B, S100A8/A9, mtDNA-CN/heteroplasmy) and imaging modalities (CMR, PET, echocardiography). The bottom panel presents therapeutic strategies (pharmacological agents, gene therapy/mitochondrial transplantation, lifestyle interventions), with an integrated bridge emphasizing the translational synergy between diagnosis and treatment. This framework aims to guide personalized risk stratification and targeted management of MI. ROS, reactive oxygen species; CMR, cardiac magnetic resonance; PET, positron emission tomography.

## 1 Introduction

Myocardial infarction (MI), commonly referred to as a heart attack, persists as a predominant cause of morbidity and mortality on a global scale. It is typically precipitated by the sudden occlusion of a coronary artery, resulting in myocardial ischemia and subsequent cardiomyocyte death due to deprivation of oxygen and nutrients. Despite advancements in reperfusion therapies and pharmacological interventions, MI continues to impose a substantial clinical burden, with survivors frequently encountering long-term complications such as heart failure, arrhythmias, and recurrent ischemic events. The pathophysiology of MI encompasses a complex cascade of events, including oxidative stress, calcium overload, inflammation, and mitochondrial dysfunction, which collectively contribute to tissue injury and adverse cardiac remodeling (1, 2).

Mitochondria, frequently referred to as the “powerhouses” of the cell, are indispensable organelles responsible for the production of adenosine triphosphate (ATP) via oxidative phosphorylation. In addition to their role in energy metabolism, mitochondria are involved in the regulation of vital cellular processes, including calcium homeostasis, reactive oxygen species (ROS) signaling, and apoptosis (3). In cardiomyocytes, which exhibit high energy requirements, the maintenance of mitochondrial integrity is crucial for preserving contractile function and cellular viability. Mitochondria contain their own circular DNA, which encodes essential subunits of the electron transport chain and is vulnerable to mutations and oxidative damage. The dynamic nature of mitochondria, characterized by processes such as fission, fusion, and mitophagy, further highlights their significance in cellular adaptation to stress (4, 5).

Recent studies have highlighted mitochondrial dysfunction as a central factor in the pathogenesis of MI. During ischemia-reperfusion (I/R) injury, mitochondria emerge as a significant source of ROS, which intensify oxidative damage to lipids, proteins, and mitochondrial DNA (mtDNA). The calcium overload-induced opening of the mitochondrial permeability transition pore (mPTP) results in the loss of membrane potential, mitochondrial swelling, and the activation of apoptotic and necrotic pathways (6). Additionally, disruptions in mitochondrial dynamics, characterized by excessive fission or insufficient fusion, undermine the integrity of the mitochondrial network, thereby exacerbating cellular injury. Genetic variations in mtDNA, including heteroplasmic mutations and altered copy number, have been associated with an increased susceptibility to MI and adverse clinical outcomes. Specifically, mutations in genes such as MT-TL1 and MT-ND5 are linked to heightened cardiovascular risk, while a reduced mtDNA copy number (mtDNA-CN) in leukocytes is an independent predictor of incident cardiovascular disease (7, 8).

Mitochondrial dysfunction plays a significant role that extends beyond the acute phase of MI. Persistent bioenergetic deficits, impaired mitochondrial biogenesis, and defective quality control mechanisms contribute to post-infarction complications, including maladaptive remodeling and the progression to heart failure (9, 10). As a result, mitochondrial components and pathways have emerged as promising diagnostic biomarkers and therapeutic targets. Specific biomarkers, such as ATP synthase F1 subunit β (ATP5B), S100A8/A9, and mitochondria-related genes like PINK1 and COX5A, hold potential for early diagnosis and risk stratification (11–14). Furthermore, therapeutic strategies aimed at preserving mitochondrial function—including pharmacological agents such as mitochondrial division inhibitor-1 (Mdivi-1), gene therapies that target mitochondrial biogenesis, and lifestyle interventions like exercise training—have demonstrated cardioprotective effects in preclinical models (15–17).

Despite significant advancements in the field, numerous controversies and challenges persist. The debate continues regarding whether mitochondrial dysfunction plays a causative or consequential role in MI (18, 19). Moreover, the translation of mitochondrial-based therapies from laboratory research to clinical practice remains challenging due to obstacles such as drug delivery issues, the translatability of experimental models, and patient heterogeneity (20). Ethical considerations in genetic and acute care research further complicate clinical applications (21, 22).

Despite increasing acknowledgment of mitochondrial dysfunction as a critical factor in MI, there remains a deficiency in standardized clinical tools for its assessment. This shortfall impedes the translation of mechanistic insights into validated diagnostic and therapeutic strategies. To address this issue, the present review systematically synthesizes current evidence to identify promising mitochondrial-based approaches, those that have not met expectations, and the underlying reasons for their shortcomings. By critically evaluating biomarkers, imaging modalities, molecular diagnostics, and therapeutic interventions, this work aims to inform the design of future clinical trials and the development of robust mitochondrial-focused biomarkers, ultimately facilitating a more precise and targeted approach to the management of MI.

## 2 Methods

### 2.1 Search strategy and data sources

This comprehensive review was conducted in accordance with the Preferred Reporting Items for Systematic Reviews and Meta-Analyses (PRISMA) guidelines. A systematic literature search was performed across three major electronic databases: PubMed, Embase, and the Cochrane Library. The search period encompassed records from inception until October 2025. The search strategy employed a combination of Medical Subject Headings (MeSH) terms and free-text keywords related to myocardial infarction and mitochondrial dysfunction. Core search terms included:

“myocardial infarction” OR “heart attack” OR “acute coronary syndrome”

“mitochondria” OR “mitochondrial dysfunction”

“diagnostics” OR “biomarker” OR “imaging”

“therapeutics” OR “treatment” OR “therapy”

“mitophagy” OR “mitochondrial biogenesis”

“heteroplasmy” OR “mtDNA”

“ischemia-reperfusion injury” OR “IRI”

Boolean operators (AND, OR) were used to combine concepts. The search was limited to articles published in English. The reference lists of relevant review articles and included studies were manually screened to identify additional pertinent publications.

### 2.2 Selection and eligibility criteria

Two independent reviewers screened titles and abstracts, followed by a full-text assessment of potentially eligible articles. Discrepancies were resolved through discussion or consultation with a third senior investigator.

Inclusion criteria were:

Studies investigating the role of mitochondrial dysfunction in the pathophysiology, diagnosis, or treatment of myocardial infarction.

Study designs including clinical trials (randomized or non-randomized), observational human studies (cohort, case-control), and meta-analyses/systematic reviews.

Preclinical studies (*in vitro* or animal models) were selectively included if they provided mechanistic insight of high translational relevance, elucidated novel pathways, or evaluated emerging therapeutic strategies with clear clinical potential.

Publication in a peer-reviewed journal.

Exclusion criteria were:

Studies not primarily focused on myocardial infarction or mitochondrial biology.

Case reports, editorials, letters, and conference abstracts without full data.

Preclinical studies with limited mechanistic novelty or unclear translational relevance to human disease.

Non-English publications.

### 2.3 Data extraction and synthesis

A standardized data extraction form was used to collect information from included studies: author(s), publication year, study design, sample characteristics (species, cell type, human population), key methodologies, primary findings related to mitochondrial dysfunction, and main conclusions. Given the heterogeneous nature of the included studies (spanning molecular mechanisms, diagnostics, and therapeutics), a narrative synthesis approach was adopted. Findings were organized thematically to address the review's objectives, covering basic mechanisms, epidemiology, diagnostics, therapeutics, controversies, and future directions.

### 2.4 Risk of bias and quality assessment

The methodological quality and risk of bias of included studies were critically appraised using established tools appropriate to each study design:

Randomized Controlled Trials (RCTs): Cochrane Risk of Bias 2 (RoB 2) tool.

Observational Studies (cohort, case-control): Newcastle-Ottawa Scale (NOS).

Preclinical Animal Studies: The SYRCLE's risk of bias tool was considered for rigor assessment, with emphasis on reporting of randomization, blinding, and sample size justification.

Systematic Reviews/Meta-Analyses: AMSTAR 2 (A MeaSurement Tool to Assess systematic Reviews 2) checklist.

This assessment informed the critical interpretation of evidence strength and the discussion of translational challenges within the manuscript.

### 2.5 PRISMA flow diagram

**Figure 1:**
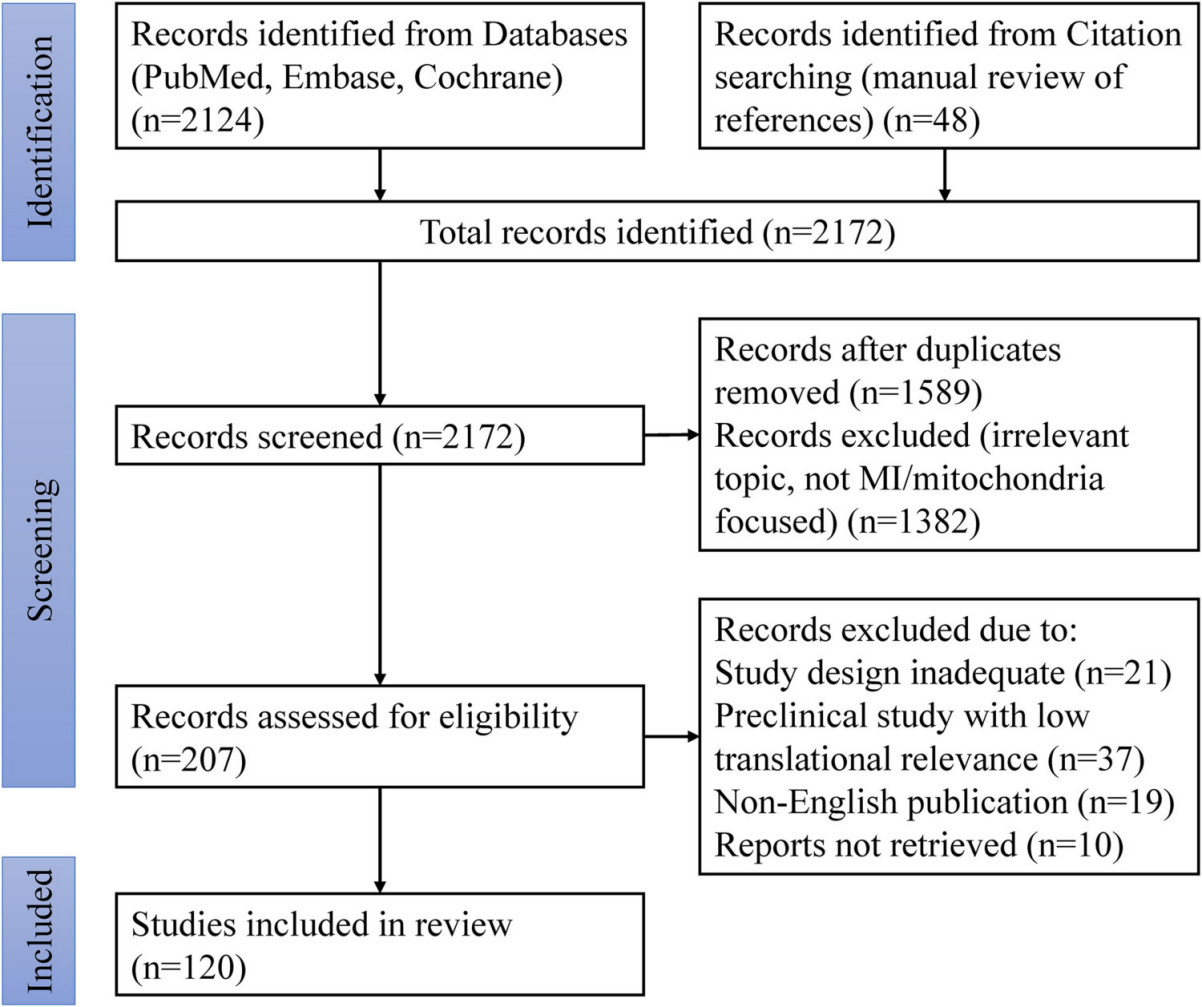
Preferred reporting items for systematic reviews and meta-analyses (PRISMA) flow diagram of study selection process. MI, Myocardial infarction.

The study selection process is summarized in the PRISMA flow diagram (Figure 1). It is important to note that while this review adheres to a systematic search and screening philosophy as outlined, the final synthesis is narrative in nature, aiming to provide a comprehensive overview rather than a statistically aggregated result. The flow diagram illustrates the scope and rigor of the literature curation process.

## 3 Mitochondrial dysfunction in MI: basic theories

### 3.1 Evolution of mitochondrial dysfunction concepts in MI

The comprehension of mitochondrial dysfunction in the context of MI has undergone substantial evolution over the years. Initially, mitochondria were predominantly perceived as the “powerhouses” of the cell, chiefly responsible for ATP production through oxidative phosphorylation. However, with the development of more sophisticated research methodologies, it has become evident that their role in MI is considerably more intricate (23, 24).

Early investigations began to acknowledge the susceptibility of mitochondria during I/R injury, a frequent consequence of MI. For instance, studies have demonstrated that pre-treatment with a single high dose of atorvastatin can confer cardioprotection in various I/R models by activating mitochondrial KATP channels (25). This discovery suggests that mitochondrial function can be modulated pharmacologically, indicating that mitochondrial dysfunction is not an unavoidable outcome but rather a process amenable to intervention.

Subsequent research has explored the molecular mechanisms underlying mitochondrial dysfunction in greater depth. It has been identified that the deletion of neuropilin-1 in cardiomyocytes and vascular smooth muscle cells leads to the induction of peroxisome proliferator-activated receptor γ coactivator 1α. This process is associated with dysregulated cardiac mitochondrial accumulation and the activation of markers related to cardiac hypertrophy and stress (26). These findings underscore the critical role of specific genes in the regulation of mitochondrial function and elucidate the complex interplay between mitochondrial homeostasis and MI.

### 3.2 Pathophysiological mechanisms of mitochondrial dysfunction in MI

Mitochondrial dysfunction in MI is intricately linked to a complex interplay of various pathophysiological mechanisms, with oxidative stress playing a pivotal role. During MI, there is an elevated production of ROS, which can inflict damage on mitochondrial components, including mtDNA, proteins, and lipids (27, 28). For example, a study investigating aging-related mitochondrial dysfunction and arrhythmia post-MI revealed that aging-associated cardiac mosaic respiratory chain dysfunction contributed to the development of both spontaneous and inducible cardiac arrhythmias following MI. This phenomenon was triggered by an increase in ROS production, resulting in mitochondrial damage and subsequent arrhythmic events (29).

**Figure 2:**
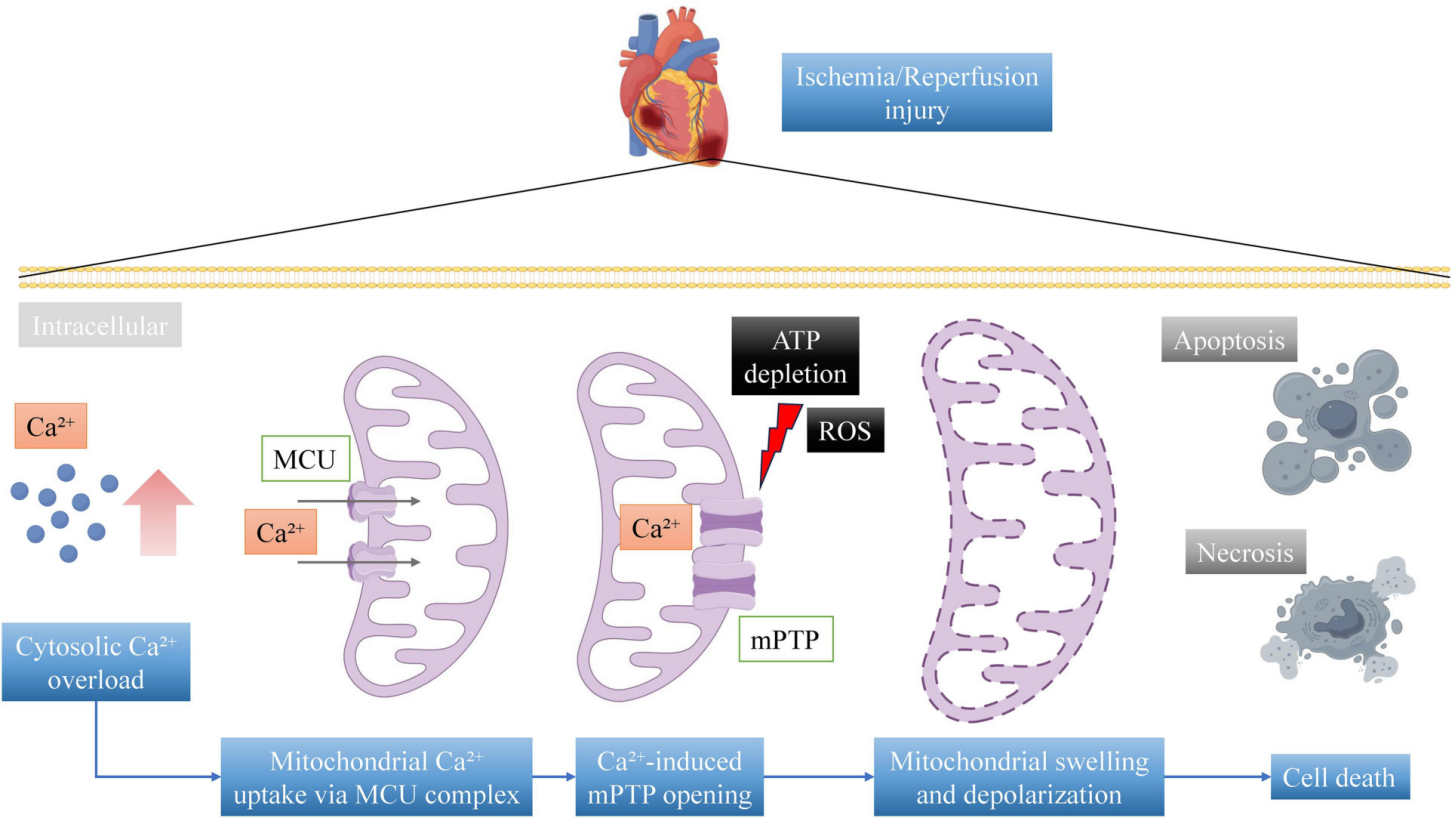
Mechanism of calcium overload-induced mitochondrial dysfunction in myocardial infarction. During ischemia-reperfusion (I/R) injury, excessive activation of calcium channels and reverse-mode operation of the Na⁺/Ca^2^⁺ exchanger (NCX) lead to cytosolic Ca^2^⁺ overload (>500 μM). The mitochondrial calcium uniporter (MCU) complex, composed of MCU, MICU1/2, and EMRE, mediates massive Ca^2^⁺ influx into the mitochondrial matrix. Elevated mitochondrial Ca^2^⁺ levels, combined with oxidative stress and ATP depletion, trigger the opening of the mitochondrial permeability transition pore (mPTP), a non-specific channel formed by cyclophilin D (CypD), adenine nucleotide translocase (ANT), voltage-dependent anion channel (VDAC), and phosphate carrier (PiC). The opening of mPTP causes mitochondrial depolarization (loss of ΔΨm), mitochondrial swelling, reactive oxygen species (ROS) burst, and ATP synthesis collapse, ultimately leading to cardiomyocyte death via apoptosis or necrosis. This figure illustrates the critical role of calcium dysregulation in driving mitochondrial dysfunction during MI.

Another significant mechanism involves the disruption of calcium homeostasis. During MI, intracellular calcium overload may occur, potentially triggering the opening of the mPTP. This calcium overload predominantly arises from dysregulated ion transport during ischemia and reperfusion. During the ischemic phase, ATP depletion inhibits the function of the sarcoplasmic/endoplasmic reticulum calcium ATPase (SERCA) and the plasma membrane calcium ATPase (PMCA), thereby impairing calcium reuptake (30). Simultaneously, intracellular acidosis activates the sodium-hydrogen exchanger (NHE), resulting in sodium accumulation, which subsequently facilitates the reverse mode operation of the sodium-calcium exchanger (NCX), leading to excessive calcium influx (31). Upon reperfusion, the abrupt restoration of oxygen and normalization of pH paradoxically exacerbate calcium overload through the rapid reactivation of NHE and NCX functions, along with uncontrolled calcium release from the sarcoplasmic reticulum via ryanodine receptors (RyR) (32–34). The activation of mPTP results in mitochondrial swelling, depolarization of the mitochondrial membrane potential, and ultimately leads to cell death (35). Studies have demonstrated that in the context of I/R injury, the introduction of calcium concentrations exceeding 500 μM to isolated mitochondria induces mitochondrial swelling, indicative of mPTP opening and calpain activation. The inhibition of calpain, a calcium-activated cysteine protease, has been shown to partially prevent mPTP opening, which underscored the critical role of calcium-mediated pathways in mitochondrial dysfunction (36). The key mechanism of calcium overload in mitochondrial dysfunction was summarized in Figure 2.

Mitochondrial dynamics, encompassing both fission and fusion processes, are critically important in cellular function. Excessive mitochondrial fission results in the formation of smaller, fragmented mitochondria, which are more susceptible to dysfunction. Conversely, impaired mitochondrial fusion can hinder the effective repair and maintenance of the mitochondrial network (37, 38). Empirical evidence from a series of studies investigating the role of mitochondrial fission in cardiac I/R injury demonstrates that the inhibition of mitochondrial fission enhances cardiac mitochondrial function, reduces infarct size, and preserves left ventricular function (18, 39). These findings underscore the necessity of maintaining a balanced mitochondrial dynamic to prevent mitochondrial dysfunction in the context of MI.

### 3.3 Role of mitochondrial genetics in MI

Mitochondrial genetics has become a pivotal field in elucidating the pathogenesis of MI. Mutations and variations in mtDNA have been linked to an elevated risk of MI (40, 41). For instance, a study examining mtDNA heteroplasmic mutations in patients with CHD and a history of MI identified mutations such as C3256T (gene MT-TL1), G12315A (gene MT-TL2), G13513A (gene MT-ND5), and G15059A (gene MT-CYB) as being significantly associated with increased cardiovascular disease risk (42).

Additionally, mtDNA-CN has been recognized as a critical factor in the pathogenesis of cardiovascular diseases (40). Research investigating the relationship between mtDNA-CN and cardiovascular disease demonstrated that a reduced mtDNA-CN, assessed in buffy coat/circulating leukocytes, was independently correlated with the incidence of cardiovascular diseases, including CHD and stroke. This finding indicates that mtDNA-CN may serve as a potential biomarker for predicting the risk of MI (43).

Furthermore, the release of mtDNA into the extracellular space has been implicated in the pathogenesis of MI (44). In cases of acute myocardial infarction (AMI), elevated levels of circulating mtDNA have been documented. Extracellular mtDNA can activate nuclear factor kappa-light-chain-enhancer of activated B cells (NF-κB) via Toll-like receptor 9, leading to cell death in cardiomyocytes and exacerbating tissue injury (45).

Additionally, genetic manipulation of genes related to mitochondrial function can significantly influence the outcomes of MI (46, 47). For instance, the overexpression of Twinkle helicase, which plays a role in mtDNA replication, has been shown to increase mtDNA-CN and ameliorate ischemic cardiomyopathy 28 days post-MI. Twinkle overexpression also significantly reduced the incidence of cardiac rupture and improved post-MI survival, accompanied by the suppression of matrix metalloproteinases MMP-2 and MMP-9 in the MI border zone (48). These findings suggest that targeting genes associated with mitochondrial function may represent a viable therapeutic strategy for MI.

### 3.4 Mitochondrial heteroplasmy in MI: concepts and clinical implications

#### 3.4.1 Definition and thresholds of heteroplasmy

Mitochondrial heteroplasmy denotes the simultaneous presence of both wild-type and mutant mtDNA within a single cell or organism. The proportion of mutant mtDNA, known as the heteroplasmy level, exhibits variability across different tissues and individuals. A critical threshold, generally between 60% and 90% mutant mtDNA, exists; surpassing this threshold leads to clinically observable mitochondrial dysfunction, impacting oxidative phosphorylation and cellular viability. This threshold is modulated by factors such as the specific mutation involved, the energy demands of the tissue, and the nuclear genetic background (49).

#### 3.4.2 Tissue-specific variation and blood vs. myocardial biopsy discrepancies

Heteroplasmy levels demonstrate significant tissue-specific variation, attributable to differences in mitotic activity, oxidative stress exposure, and mitochondrial turnover. In the context of MI, cardiac muscle—a post-mitotic tissue with high energy demands—may accumulate higher levels of mutant mtDNA over time compared to tissues with rapid cellular turnover, such as blood leukocytes. As a result, heteroplasmy levels assessed in peripheral blood may not accurately represent the mtDNA mutation burden within the myocardium, potentially leading to discrepancies between blood-based genetic screening and the actual mitochondrial status of cardiac tissue. Current research indicates that myocardial biopsy remains the definitive method for evaluating cardiac-specific heteroplasmy. However, non-invasive imaging techniques and circulating biomarkers are being developed to address this discrepancy (50, 51).

#### 3.4.3 Impact on MI susceptibility and prognosis

Elevated levels of heteroplasmy in cardiac tissue have been associated with an increased susceptibility to MI and poorer clinical outcomes. Specifically, heteroplasmic mutations in genes such as *MT-TL1* (e.g., C3256 T) and *MT-ND5* (e.g., G13513A) are linked to compromised mitochondrial respiration, heightened production of ROS, and an enlarged infarct size following I/R injury. Furthermore, patients exhibiting high heteroplasmy loads demonstrate reduced recovery of left ventricular function, a higher incidence of post-MI heart failure, and an elevated risk of arrhythmias. These findings highlight the prognostic significance of assessing heteroplasmy levels in stratifying MI patients for targeted monitoring and therapeutic interventions (52).

#### 3.4.4 Future directions in heteroplasmy research

Emerging technologies, including single-cell mtDNA sequencing, digital droplet PCR, and long-read sequencing, are significantly advancing our capacity to detect low-level heteroplasmy and quantify tissue-specific mutation loads. In addition, gene-editing methodologies, such as CRISPR-based mitochondrial editing, alongside heteroplasmy-shifting agents like mitochondrial-targeted nucleases, offer promising therapeutic strategies aimed at reducing the burden of mutant mtDNA and enhancing cardiac outcomes. To facilitate the translation of these advancements into clinical practice, there is a critical need for standardized protocols for heteroplasmy quantification and validation across multi-center cohorts (53).

### 3.5 Mitochondrial epigenetics and methylation in MI

#### 3.5.1 mtDNA methylation: mechanisms and functional implications

Mitochondrial epigenetics, particularly methylation of mtDNA, has emerged as a critical regulatory layer in mitochondrial function and dysfunction. Unlike nuclear DNA, mtDNA is circular, lacks histones, and is organized into protein–DNA complexes called nucleoids. Nevertheless, mtDNA can undergo epigenetic modifications, primarily methylation at CpG and non-CpG sites, which influence gene expression, replication, and stability (54, 55).

Methylation of the D-loop region, a key regulatory area for mtDNA replication and transcription, has been associated with altered mitochondrial gene expression and respiratory chain function. For instance, hypermethylation of the D-loop has been linked to reduced mtDNA-CN and impaired oxidative phosphorylation in cardiovascular diseases. In the context of MI, ischemia-induced oxidative stress can modify DNA methyltransferase activity, leading to aberrant mtDNA methylation patterns that exacerbate mitochondrial dysfunction (56, 57).

#### 3.5.2 Mitochondrial nucleoids: structural and regulatory roles

Mitochondrial nucleoids are compact structures comprising mtDNA and associated proteins such as TFAM (mitochondrial transcription factor A), POLG (DNA polymerase gamma), and mtSSB (mitochondrial single-stranded binding protein). These nucleoids regulate mtDNA packaging, transcription, and segregation. Post-MI, nucleoid integrity is compromised due to oxidative damage and proteolytic stress, leading to mtDNA release and activation of inflammatory pathways. TFAM, in particular, plays a dual role in maintaining mtDNA stability and modulating methylation patterns by recruiting DNA methyltransferases to specific mtDNA regions (58).

#### 3.5.3 Cross-talk with the nuclear epigenome

Mitochondrial and nuclear genomes engage in continuous bidirectional communication to coordinate cellular energy metabolism and stress responses. This cross-talk is mediated by retrograde signaling pathways, including calcium signaling, ROS, and NAD+/NADH ratios, which influence nuclear epigenetic modifiers such as histone deacetylases (HDACs), DNA methyltransferases (DNMTs), and ten-eleven translocation (TET) enzymes (59).

In MI, mitochondrial dysfunction alters the nuclear epigenetic landscape, affecting the expression of genes involved in inflammation, apoptosis, and metabolism. Conversely, nuclear epigenetic changes can modulate mitochondrial biogenesis and dynamics by regulating transcription factors such as PGC-1α, NRF1, and TFAM. For example, hypermethylation of the PGC-1α promoter has been observed in post-MI hearts, leading to reduced mitochondrial biogenesis and persistent bioenergetic deficit (60).

#### 3.5.4 Mitochondrial epigenetic marks as diagnostic biomarkers post-MI

Given their stability and detectability in peripheral blood, mitochondrial epigenetic marks hold promise as non-invasive biomarkers for MI diagnosis, risk stratification, and prognosis. Altered mtDNA methylation levels in the D-loop region have been reported in patients with coronary artery disease and MI, correlating with infarct size and left ventricular dysfunction. Furthermore, changes in TFAM expression and nucleoid morphology may serve as indicators of mitochondrial stress and recovery potential post-MI (22).

Emerging technologies, such as bisulfite sequencing and methylation-sensitive PCR, now enable high-resolution mapping of mtDNA methylation patterns. Integrating these epigenetic markers with traditional biomarkers like troponin and mitochondrial genes (e.g., PINK1, COX5A) could enhance diagnostic accuracy and guide personalized therapeutic interventions (54, 61).

#### 3.5.5 Future directions and clinical potential

Despite its promise, mitochondrial epigenetics remains an understudied field in cardiovascular medicine. Future research may focus on:

1. Elucidating the dynamic changes in mtDNA methylation during the acute and chronic phases of MI.
2. Investigating tissue-specific methylation patterns and their correlation with clinical outcomes.
3. Exploring the therapeutic potential of modulating mitochondrial epigenetics through pharmacological agents or lifestyle interventions.

Understanding the interplay between mitochondrial epigenetics and nuclear regulation will be essential for developing integrated diagnostic and therapeutic strategies for MI patients.

### 3.6 Genetic insights into mitochondrial function: from GWAS to multi-omic integration

While the role of mitochondrial genetics, such as mtDNA mutations and copy number variations, is well-established, the application of traditional genome-wide association studies (GWAS) to understand mitochondrial dysfunction in MI has faced significant challenges. However, emerging methodologies are beginning to overcome these limitations, offering deeper insights into the complex genetic architecture governing mitochondrial health in the heart.

#### 3.6.1 Limitations of traditional GWAS for mitochondrial traits in MI

Traditional GWAS, which scan the nuclear genome for associations with disease risk, have had limited success in identifying robust signals related to mitochondrial function in MI for several reasons. First, the majority of mitochondrial proteins are nuclear-encoded, and the effects of common nuclear variants on these genes are often small and diffuse, making them difficult to detect against the backdrop of polygenic risk for MI itself (62, 63). Second, these studies rarely account for the unique characteristics of the mitochondrial genome. mtDNA is present in multiple copies per cell, exhibits heteroplasmy, and is maternally inherited, complexities that standard GWAS pipelines are not designed to handle. Consequently, associations with specific mtDNA haplogroups or heteroplasmic variants have often been missed or underpowered in large-scale cohorts (64). The inability to simultaneously analyze both nuclear and mitochondrial genomes in a unified model has thus obscured the dual genetic control of mitochondrial function.

#### 3.6.2 Emerging approaches: mitoGWAS and mt-eQTLs

To address these gaps, the field is moving towards specialized “mitoGWAS” and the mapping of mitochondrial expression quantitative trait loci (mt-eQTLs). MitoGWAS represents a refined analytical strategy that explicitly models both nuclear and mitochondrial genomic data. Instead of treating the mitochondrial genome as a static entity, these approaches incorporate mtDNA haplogroups, copy number, and even heteroplasmy levels as quantitative traits or covariates in the association analysis with MI (65, 66). This allows for the detection of interplay between the two genomes, revealing, for example, how a specific nuclear background might modify the phenotypic expression of a particular mtDNA variant.

Complementing this, the identification of mt-eQTLs provides a functional bridge from genetic variation to molecular phenotype. Mt-eQTLs are nuclear genetic variants that influence the expression levels of both nuclear-encoded mitochondrial genes and, intriguingly, the transcription of mtDNA itself (67). By integrating large-scale transcriptomic data from cardiac tissues with genomic data, researchers can pinpoint regulatory variants that modulate the expression of key mitochondrial complexes. For instance, a variant in a gene encoding a mitochondrial transcription factor could act as a trans-mt-eQTL, affecting the expression of mtDNA-encoded genes and ultimately impacting oxidative phosphorylation capacity (68). These approaches move beyond simple disease association to uncover the regulatory mechanisms that control mitochondrial abundance and function.

#### 3.6.3 Future directions: integrative multi-omic strategies

The future of genetic discovery in this field lies in integrative multi-omic strategies. This involves moving beyond genomics and transcriptomics to incorporate proteomics, metabolomics, and epigenomic data. For example, integrating proteomic data (like the levels of ATP5B or S100A8/A9) with genomic data can help validate the functional impact of a genetic variant and identify protein biomarkers that mediate the genetic risk (12, 69). Similarly, metabolomic profiling can reveal downstream consequences of genetically driven mitochondrial dysfunction, such as altered levels of specific acylcarnitines or TCA cycle intermediates, which may serve as functional readouts of variant pathogenicity (70).

Furthermore, single-cell technologies are poised to revolutionize this area. Single-cell sequencing can resolve the cellular heterogeneity of the heart, allowing researchers to ask whether the effect of a particular mtDNA heteroplasmy or nuclear risk allele is cell-type specific (e.g., affecting cardiomyocytes more than fibroblasts) (71). This level of resolution is critical for understanding the precise mechanisms by which genetic variation leads to mitochondrial dysfunction and ultimately to MI and its post-infarction complications. By embracing these integrative and high-resolution approaches, the field is finally poised to unlock the full genetic architecture of mitochondrial dysfunction in MI, paving the way for truly personalized risk stratification and targeted therapies.

## 4 Epidemiology of mitochondrial dysfunction in MI

### 4.1 Prevalence of mitochondrial dysfunction in MI patients

The prevalence of mitochondrial dysfunction among patients with MI has garnered increasing attention in recent research. Empirical evidence indicates that mitochondrial dysfunction is a prevalent phenomenon in these patients, playing a crucial role in the disease's pathophysiology (22). Mitochondrial impairment is observed across various stages of MI, from acute I/R injury to chronic post-infarction remodeling, and is increasingly recognized as a hallmark of myocardial injury.

A study investigating the impact of estrogen supplementation on antioxidant enzymes and mitochondrial respiratory function in ovariectomized post-MI rats revealed that ovariectomy exacerbates mitochondrial dysfunction following MI. This finding suggests that hormonal factors may modulate mitochondrial function in the context of MI, potentially leading to a higher prevalence of mitochondrial dysfunction in specific patient demographics, such as postmenopausal women (72). Additionally, clinical studies have reported that up to 60%–70% of patients with ST-elevation MI exhibit measurable mitochondrial respiratory chain impairment in circulating leukocytes, correlating with infarct size and adverse outcomes (43).

Another study investigating the role of S100a8/a9 in myocardial I/R injury revealed that S100a8/a9, identified as the most significantly upregulated gene during the early reperfusion phase, induces mitochondrial respiratory dysfunction in cardiomyocytes. Notably, serum levels of S100a8/a9 were markedly elevated one day following percutaneous coronary intervention in patients experiencing AMI, indicating that mitochondrial dysfunction occurs early in the disease progression (69). Moreover, elevated levels of circulating mtDNA, a marker of mitochondrial damage, have been consistently detected in AMI patients, with higher levels associated with greater infarct size and higher risk of heart failure (45).

Additionally, research examining the association between mtDNA-CN and the incidence of heart failure in middle-aged women demonstrated that a lower baseline mtDNA-CN correlates with a higher incidence of heart failure. Given that MI is a significant risk factor for heart failure, these findings suggest that mitochondrial dysfunction, as evidenced by reduced mtDNA-CN, may be prevalent among MI patients and contribute to the subsequent development of heart failure (73). Furthermore, a recent proteomic study identified significant downregulation of mitochondrial complex I and V subunits in plasma from AMI patients within 3 h of symptom onset, underscoring the early and widespread nature of mitochondrial disruption (12).

Collectively, these studies highlight that mitochondrial dysfunction is not merely a secondary phenomenon but a prevalent and early event in MI, detectable through multiple biomarkers and functional assays, and significantly influences clinical progression and prognosis.

### 4.2 Demographic variations in mitochondrial dysfunction and MI

Demographic variations significantly influence the relationship between mitochondrial dysfunction and MI. Age-stratified, sex-related differences have been documented in the incidence, management, and outcomes of AMI (74, 75). A study examining the impact of female sex on MI across different age groups revealed that the incidence rate of hospitalizations for MI was consistently lower in women than in men across all age categories. However, post-MI outcomes exhibited age-specific patterns. The adjusted odds of mortality for women, compared to men, varied by age, with the adverse effects of female sex on most outcomes being most pronounced in young and middle-aged women (76).

The underlying mechanisms contributing to these differences may be attributed to hormonal factors, lifestyle variations, and comorbidities. For instance, premenopausal women may experience a lower incidence of MI due to the cardioprotective effects of estrogen. However, postmenopausal women may face an increased risk of mitochondrial dysfunction and subsequent MI due to the loss of estrogen's protective effects (77). Additionally, disparities in the prevalence of risk factors such as hypertension, diabetes, and obesity between men and women, along with differences in healthcare access and utilization, may further contribute to these demographic variations. Furthermore, age is a significant factor. Aging is associated with an increased prevalence of mitochondrial dysfunction, as evidenced by studies on aging-related mitochondrial dysfunction and arrhythmia following MI (78, 79). Older individuals may exhibit a heightened susceptibility to MI-related mitochondrial dysfunction due to age-related alterations in mitochondrial function, including increased oxidative stress and reduced mitochondrial biogenesis (29).

### 4.3 Risk factors linking mitochondrial dysfunction to MI

Numerous risk factors have been identified that establish a connection between mitochondrial dysfunction and MI. Among these, hypertension emerges as a particularly significant risk factor. The association of oxidative stress with hypertension can precipitate mitochondrial dysfunction (80). The enzymatic activation of NADPH oxidase and xanthine oxidase, along with the uncoupling of endothelial nitric oxide synthase (eNOS) and mitochondrial dysfunction within the vascular wall, culminates in the generation of superoxide anion (81). This oxidative stress condition results in a diminished bioavailability of nitric oxide and prostacyclin, thereby contributing to the pathogenesis of MI (82).

Diabetes constitutes a significant risk factor, particularly in the context of diabetic cardiomyopathy, where factors such as impaired calcium handling, increased oxidative stress, and mitochondrial dysfunction play critical roles. Mitochondrial dysfunction associated with diabetes can result in altered metabolism, elevated ROS production, and subsequent myocardial injury (83, 84). Furthermore, various microRNAs implicated in the pathogenesis of diabetic cardiomyopathy have also been linked to cardiovascular diseases such as MI, underscoring the intricate relationship between diabetes, mitochondrial dysfunction, and MI (85).

Physical inactivity and sedentary lifestyle are recognized as significant risk factors. Research utilizing mouse models has demonstrated that physical inactivity, when combined with MI, exacerbates the decline in survival rates, induces cardiac hypertrophy, increases mitochondrial fission in cardiomyocytes, and activates myofibroblasts, leading to cardiac fibrosis. These findings suggest that mitochondrial fission may contribute to the detrimental effects of physical inactivity on MI through abnormal interactions between cardiomyocytes and fibroblasts (86).

Moreover, various factors, including obesity, dyslipidemia, and smoking, are implicated in mitochondrial dysfunction and heightened MI risk. Obesity is associated with chronic low-grade inflammation and increased oxidative stress, which impair mitochondrial respiration and promote excessive mitochondrial fission, thereby compromising cardiac bioenergetics and increasing susceptibility to ischemic injury (87). Dyslipidemia, particularly elevated circulating free fatty acids and oxidized low-density lipoprotein, leads to intramyocardial lipid accumulation and subsequent lipotoxicity. This process disrupts mitochondrial membrane integrity, inhibits fatty acid β-oxidation, and enhances ROS production through electron transport chain uncoupling, collectively contributing to mitochondrial dysfunction and cardiomyocyte apoptosis (88). Smoking, through its numerous toxic constituents such as nicotine and reactive aldehydes, induces direct oxidative damage to mitochondrial proteins and mtDNA, while also promoting systemic inflammation that further exacerbates mitochondrial impairment and endothelial dysfunction, thereby accelerating atherosclerotic progression and plaque instability (89, 90).

## 5 Diagnostic techniques for mitochondrial dysfunction in MI

### 5.1 Biomarkers for mitochondrial dysfunction in MI

Biomarkers are integral to diagnosing mitochondrial dysfunction in MI. ATP5B has been identified as a potential biomarker in this context (91). Utilizing an isobaric tags for relative and absolute quantitation-based proteomics approach in a beagle dog MI model, researchers observed a significant decrease in ATP5B expression during the early stages of MI. This finding suggests that mitochondrial dysfunction and electron transport chain disruption are critical indicators of early MI, detectable within three hours (12) (Table 1).

**Table 1:** Biomarkers of mitochondrial dysfunction in myocardial infarction.

Biomarker	Source/sample type	Omics platform/detection method	Clinical utility	Current limitations/challenges
mtDNA copy number (mtDNA-CN)	Peripheral blood leukocytes, plasma	qPCR, sequencing	Predicts incident CVD, HF, and post-MI mortality	Tissue specificity (blood vs. heart); lack of standardized cutoffs
Mitochondrial heteroplasmy (e.g., MT-TL1, MT-ND5)	Blood, myocardial tissue	NGS, digital droplet PCR	Associated with MI susceptibility and adverse outcomes	Requires tissue-specific assessment; technical standardization needed
ATP5B	Plasma	Proteomics (iTRAQ)	Decreased in early MI (<3 h); reflects complex V integrity	Early-stage validation; limited large-scale studies
S100A8/A9	Serum	Immunoassay	Upregulated in early reperfusion; correlates with MACE	Reflects inflammation-induced dysfunction; not mitochondria-specific
PINK1, COX5A, TACO1	Blood, cardiac tissue	qPCR, transcriptomics	Reduced expression in AMI; associated with mitochondrial quality control	Primarily transcriptomic; lacks protein-level validation
GDF-15	Plasma	Immunoassay	Marker of mitochondrial stress; prognostic in CVD	Not specific to MI; elevated in multiple pathologies
Circulating cell-free mtDNA	Plasma	qPCR, digital PCR	Activates TLR9/NF-κB; correlates with infarct size	Pre-analytical variability; source (tissue vs. blood) unclear

Additionally, S100a8/a9 has been recognized as a significant biomarker. Studies have demonstrated that S100a8/a9 is markedly upregulated during the early reperfusion phase of myocardial I/R injury, contributing to mitochondrial respiratory dysfunction in cardiomyocytes (69, 92). Furthermore, elevated serum levels of S100a8/a9 have been observed in patients with AMI following percutaneous coronary intervention, with these elevated levels correlating with the incidence of major adverse cardiovascular events (69).

MtDNA-related biomarkers have garnered significant interest in recent research. Alterations in mtDNA, including mutations, changes in copy number, and variations in haplogroups, have been linked to the dysregulated expression of the oxidative phosphorylation system, ultimately leading to mitochondrial dysfunction (93). For instance, a study investigating mtDNA alterations in CHD suggested that mtDNA defects may be pivotal for early diagnosis, the identification of disease-specific biomarkers, and the prediction of outcomes in patients with atherosclerosis and CHD (94). Additionally, several genes associated with mitochondrial function, such as the translational activator of cytochrome c oxidase I (TACO1), cytochrome c oxidase subunit Va (COX5A), PTEN-induced putative kinase 1 (PINK1), SURF1, and NDUFA11, have been identified as critical mitochondria-related genes in AMI (95, 96). The expression levels of these genes are significantly reduced in the blood of AMI patients compared to healthy individuals, suggesting their potential utility as biomarkers for diagnosing AMI and predicting major adverse cardiovascular events (97).

### 5.2 Imaging techniques for assessing mitochondrial dysfunction in MI

**Figure 3:**
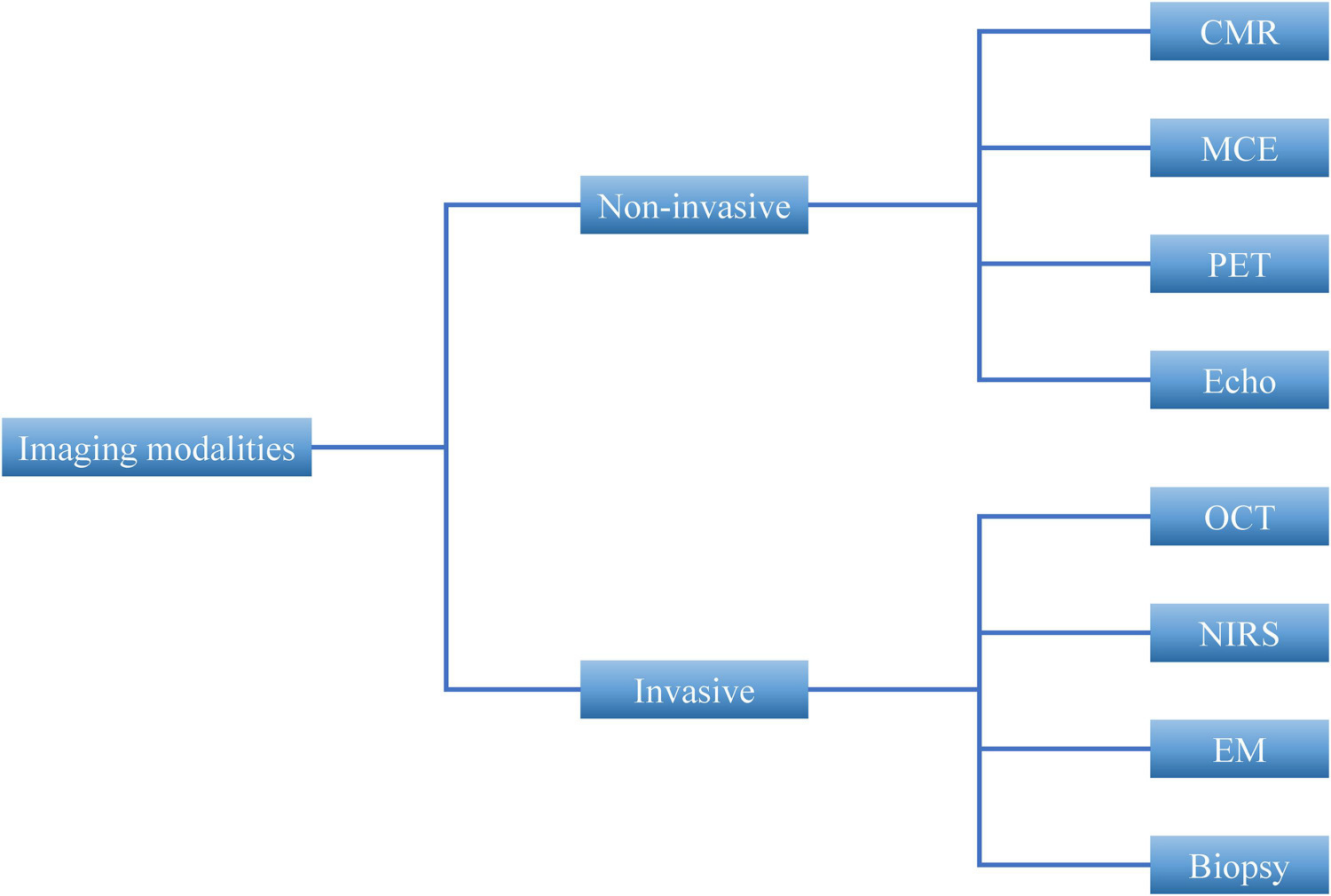
Imaging modalities for assessing mitochondrial dysfunction in myocardial infarction. CMR, cardiac magnetic resonance; MCE, myocardial contrast echocardiography; PET, positron emission tomography; Echo, Echocardiography; OCT, optical coherence tomography; NIRS, near-infrared spectroscopy; EM, electron microscopy; Biopsy, Endomyocardial biopsy.

Imaging modalities have emerged as essential tools for evaluating mitochondrial dysfunction in the context of MI (98). Based on their invasiveness, these techniques can be broadly classified into non-invasive and invasive methods (Figure 3 and Table 2). Currently, non-invasive imaging is the mainstay for clinical assessment, whereas invasive approaches remain largely investigational or are used in selected research settings.

**Table 2:** Diagnostic modalities for assessing mitochondrial dysfunction in myocardial infarction.

Modality	Invasiveness	Key parameters/target	Correlation with mitochondrial function/pathology	Clinical utility & stage
Cardiac Magnetic Resonance (CMR)	Non-invasive	Infarct size, microvascular obstruction, myocardial edema	Infarct size correlates with respiratory chain impairment; MVO reflects sustained ischemia	Established prognostic tool; strong predictor of MACE
Positron Emission Tomography (PET)	Non-invasive	Glucose metabolism (FDG), mitochondrial membrane potential (novel tracers)	FDG uptake reflects inflammation and metabolic shift post-MI; novel tracers enable direct mitochondrial assessment	Established for inflammation; investigational for direct mitochondrial imaging
Myocardial Contrast Echocardiography (MCE)	Non-invasive	Microvascular perfusion defect	Perfusion defect area indicates ischemic tissue burden and secondary mitochondrial injury	Moderate correlation with LVEF; predicts functional recovery
Speckle-Tracking Echocardiography	Non-invasive	Global longitudinal strain (GLS)	GLS parallels *ex vivo* mitochondrial bioenergetic deficits	Sensitive marker of subclinical dysfunction; independent prognostic value
Endomyocardial Biopsy + Electron Microscopy	Invasive	Mitochondrial ultrastructure (cristae, swelling, matrix density)	Direct visualization of mitochondrial structural damage	Gold standard for structural assessment; limited by sampling error and invasiveness
Seahorse XF Assay (research only)	*Ex vivo* (surrogate cells/tissue)	Oxygen consumption rate (OCR), spare respiratory capacity	Measures oxidative phosphorylation and mitochondrial reserve	Preclinical/translational use only; lacks clinical standardization

#### 5.2.1 Non-invasive imaging methods

##### 5.2.1.1 Cardiac magnetic resonance (CMR)

CMR is extensively utilized due to its ability to provide comprehensive information on myocardial structure and function. It quantifies infarct size, transmural necrosis, myocardial edema, and microvascular obstruction (including intramyocardial hemorrhage and the no-reflow phenomenon) (99). These parameters are directly linked to mitochondrial injury: infarct size correlates strongly with the extent of mitochondrial respiratory chain impairment, and microvascular obstruction reflects sustained ischemia that perpetuates mitochondrial damage. Correlation with heart function: Infarct size measured by late gadolinium enhancement (LGE) shows a strong inverse correlation with left ventricular ejection fraction (LVEF) (r typically −0.6 to −0.8) and is a well-validated predictor of adverse remodeling. Prognostic value: In post-MI patients, CMR-derived infarct size and microvascular obstruction are independent predictors of major adverse cardiovascular events (MACE), including heart failure hospitalization and mortality (99, 100).

##### 5.2.1.2 Myocardial contrast echocardiography (MCE)

MCE assesses myocardial perfusion at the microvascular level. During the subacute phase of ST-elevation MI, the extent of the perfusion defect corresponds to areas of both myocardial and microvascular necrosis, providing indirect evidence of mitochondrial dysfunction secondary to prolonged ischemia (101). Correlation with heart function: The size of the perfusion defect correlates moderately with regional wall motion abnormalities and global LVEF (r ≈ −0.5). Prognostic value: Persistent perfusion defects on MCE predict poor functional recovery and adverse left ventricular remodeling at 6–12 months, and are associated with an increased risk of heart failure (101).

##### 5.2.1.3 Positron emission tomography (PET)

PET, often combined with CT or CMR, enables metabolic and inflammatory imaging. After MI, increased myocardial glucose utilization (detected by FDG-PET) reflects inflammatory cell infiltration and altered cardiomyocyte metabolism, both linked to mitochondrial dysfunction (100). Correlation with heart function: The intensity and extent of FDG uptake in the infarct zone show a modest correlation with LVEF (r ≈ −0.4) and are associated with larger infarct size. Prognostic value: Elevated FDG signal early after MI has been associated with a higher incidence of MACE, possibly because it denotes active tissue damage and oxidative stress (100). Novel PET tracers targeting mitochondrial membrane potential (e.g., 18F-BMS-747158-02) are under investigation and may offer direct quantification of mitochondrial function *in vivo*.

#### 5.2.2 Echocardiography (conventional and strain imaging)

While conventional echocardiography is routinely used to assess LVEF and wall motion, advanced techniques like speckle-tracking echocardiography (global longitudinal strain, GLS) provide sensitive measures of subclinical dysfunction. In preclinical studies, alterations in GLS paralleled mitochondrial bioenergetic deficits measured *ex vivo* (102). Correlation with heart function: GLS correlates closely with LVEF (*r* ≈ 0.7–0.8) and is more sensitive than LVEF in detecting early myocardial impairment. Prognostic value: In MI survivors, impaired GLS is an independent predictor of all-cause mortality and heart failure, although its direct link to mitochondrial dysfunction in humans requires further validation (102).

#### 5.2.3 Invasive imaging methods

Direct assessment of mitochondrial morphology and function within the myocardium requires tissue sampling. Invasive imaging techniques, such as optical coherence tomography (OCT) or near-infrared spectroscopy (NIRS) during cardiac catheterization, are primarily used to evaluate coronary plaque characteristics rather than mitochondrial status. Electron microscopy of endomyocardial biopsy specimens remains the gold standard for visualizing mitochondrial ultrastructure (e.g., cristae remodeling, swelling, matrix density), but its invasive nature, sampling error, and lack of real-time functional data limit routine clinical use. Consequently, no invasive imaging method is currently employed to directly quantify mitochondrial function in MI patients.

Among non-invasive techniques, CMR and PET offer the strongest correlations with mitochondrial injury and cardiac function, and they carry established prognostic value. MCE and advanced echocardiography provide complementary information on perfusion and mechanics. Invasive methods are reserved for research or when tissue diagnosis is mandatory. The integration of these imaging modalities with circulating biomarkers and molecular diagnostics holds promise for a multi-parametric assessment of mitochondrial health, enabling better risk stratification and monitoring of mitochondria-targeted therapies.

### 5.3 Advances in molecular diagnostics for mitochondrial dysfunction in MI

Recent advancements in molecular diagnostics have significantly enhanced our understanding of mitochondrial dysfunction in the context of MI. Next-generation sequencing (NGS) has been instrumental in elucidating the molecular underpinnings of numerous primary mitochondrial diseases, which may have implications for mitochondrial dysfunction associated with MI (103). Despite the limitations inherent in current sequencing methodologies, advances in long-read mtDNA sequencing and NGS-based discovery of nuclear modifiers pave the way for overcoming these challenges in mitochondrial research (104).

Additionally, proteomic analyses have been employed to identify critical molecules implicated in mitochondrial dysfunction during MI (12, 105). For instance, a study investigating the role of serine carboxypeptidase 1 (Scpep1) in MI utilized proteomic analysis to uncover its downstream functional mediators. The findings revealed that Scpep1 exacerbates MI-induced cardiac dysfunction and damage by impairing mitochondrial bioenergetics through its interaction with Pex3, leading to Pex3 degradation and consequently promoting mitochondrial fission and apoptosis (106).

Molecular imaging techniques, particularly those employing targeted imaging agents, facilitate the visualization of the molecular processes that govern the immune cell response and subsequent remodeling following MI (107, 108). These methodologies offer valuable insights into mitochondrial-related processes; however, the challenge remains in effectively translating these findings into cost-efficient and clinically beneficial applications in patient care (109).

Moreover, the identification of non-coding RNAs (ncRNAs) that regulate mitochondrial functions has emerged as a significant area of research. NcRNAs, including microRNAs, long non-coding RNAs, and circular RNAs, play a role in the progression of cardiovascular diseases by influencing mitochondrial dynamics and modulating genes and signaling pathways associated with mitochondrial function (110, 111). For instance, specific microRNAs such as miR-210, miR-34a, and miR-499 have been implicated in MI pathogenesis by regulating mitochondrial metabolism, apoptosis, and oxidative stress response. Long non-coding RNAs like H19, MALAT1, and MEG3 are involved in modulating mitochondrial biogenesis, fission/fusion balance, and cardiomyocyte survival post-MI. Circular RNAs, including circNCX1 and circHIPK3, have also been reported to interact with mitochondrial proteins and affect energy metabolism and cell death pathways following ischemic injury. Certain ncRNAs may serve as promising diagnostic and prognostic biomarkers, as well as potential therapeutic targets for patients with MI (112).

### 5.4 Functional assays for mitochondrial function: the case of seahorse XF and clinical limitations

In addition to biomarker quantification and imaging modalities, functional assays that directly measure mitochondrial bioenergetics have significantly advanced preclinical research into MI. Among these, the Seahorse XF (Extracellular Flux) analyzer has emerged as a powerful tool for assessing key parameters of mitochondrial function, including oxygen consumption rate (OCR, a measure of oxidative phosphorylation) and extracellular acidification rate (ECAR, a measure of glycolysis), in real time. By utilizing specific metabolic modulators (e.g., oligomycin, FCCP, rotenone/antimycin A), the Seahorse platform can delineate critical aspects of mitochondrial physiology such as basal respiration, ATP-linked respiration, maximal respiratory capacity, and spare respiratory capacity (103, 113). This technology has been instrumental in elucidating the mechanistic pathways of mitochondrial dysfunction in isolated cardiomyocytes, cardiac tissues, and even in immune cells from preclinical models of I/R injury (113).

However, despite its significant contributions to fundamental research, Seahorse XF technology is not considered a gold standard or a routine tool in clinical diagnostics for mitochondrial dysfunction in MI. This limitation arises from several inherent and practical challenges that currently preclude its application for patient-level decision-making.

1. Sample Requirements and Tissue Accessibility: Seahorse assays typically require a large number of viable, high-quality cells or freshly isolated mitochondria. In the clinical setting of AMI, obtaining a sufficient quantity of viable cardiomyocytes is virtually impossible without an invasive endomyocardial biopsy, a procedure with inherent risks and limited clinical utility in the acute phase. While surrogate cells, such as circulating peripheral blood mononuclear cells (PBMCs) or platelets, can be obtained less invasively, the extent to which their mitochondrial respiratory profile accurately reflects the bioenergetic state of the stressed or injured myocardium remains a significant concern (114). This discrepancy between surrogate and target tissues represents a major obstacle to clinical translation.
2. *Ex Vivo* Artifact and Loss of Physiological Context: The measurement of mitochondrial function using Seahorse is performed *ex vivo* under standardized, yet artificial, conditions. Cells are removed from their complex *in vivo* environment, which includes neurohormonal influences, dynamic substrate availability, and mechanical stressors that profoundly shape mitochondrial activity. The isolation and plating procedures themselves can induce cellular stress, potentially altering the very metabolic parameters being measured. Consequently, the bioenergetic profile obtained *ex vivo* may not accurately represent the dynamic and integrated function of mitochondria within the intact, beating heart (115, 116).
3. Cellular Heterogeneity and Interpretation Complexity: Mitochondrial function is inherently heterogeneous across different cell types within the myocardium (e.g., cardiomyocytes, fibroblasts, endothelial cells, resident immune cells). In assays using tissue homogenates or mixed cell populations like PBMCs, the resulting OCR measurement represents an average of potentially diverse mitochondrial states, obscuring cell-type-specific contributions to dysfunction. Furthermore, the interpretation of Seahorse data is complex and requires rigorous normalization to cell count, protein content, or mitochondrial mass. Without such normalization, differences in OCR could simply reflect differences in cell number or mitochondrial density rather than true functional differences per mitochondrion (117, 118).
4. Lack of Standardization for Clinical Use: For a diagnostic test to be clinically actionable, it must be standardized, reproducible, and have well-defined reference ranges. Currently, Seahorse protocols, from cell isolation and plating densities to assay conditions and data normalization strategies, vary considerably across laboratories. This lack of standardization precludes the establishment of universal reference values for “normal” or “dysfunctional” mitochondrial respiration that could be applied to individual patients. The intra- and inter-individual variability, influenced by factors such as age, sex, circadian rhythms, and comorbidities, further complicates the definition of a pathological threshold (119, 120).

In summary, while Seahorse XF analysis remains an indispensable tool for dissecting mitochondrial mechanisms in preclinical models and for hypothesis generation, its current methodological and biological limitations render it unsuitable as a routine clinical diagnostic for mitochondrial dysfunction in MI. Its future clinical utility may depend on the development of standardized, minimally invasive protocols and a more sophisticated understanding of how *ex vivo* measurements in surrogate cells correlate with true myocardial bioenergetic status.

### 5.5 The translational gap of diagnostic kit

Despite the well-established role of mitochondrial dysfunction in the pathogenesis and progression of MI, and the plethora of potential biomarkers identified in preclinical studies, there is currently no clinically approved diagnostic kit specifically designed to assess mitochondrial health for MI diagnosis, risk stratification, or therapeutic guidance. This translational gap stems from a convergence of biological, technical, and regulatory barriers (22).

#### 5.5.1 Barriers to clinical translation

1. Biological Complexity and Lack of Specificity: Mitochondrial dysfunction is not a monolithic entity but a heterogeneous state involving multiple pathways (e.g., bioenergetic failure, oxidative stress, impaired dynamics, mtDNA damage). Single biomarkers, such as mtDNA-CN or circulating S100A8/A9, while statistically associated with MI in population studies, lack the specificity and sensitivity required for individual patient-level decision-making. Their levels can be influenced by numerous confounding factors including age, sex, comorbidities (e.g., diabetes, hypertension), and medications, making it difficult to establish a clear, universal diagnostic threshold (121).
2. Tissue Specificity and Surrogate Sample Limitations: The ideal sample for assessing myocardial mitochondrial health is cardiac tissue itself, which is inaccessible without invasive biopsy. Surrogate tissues, such as peripheral blood mononuclear cells (PBMCs) or platelets, are easily obtainable but may not accurately reflect the bioenergetic state of the stressed or injured myocardium. The correlation between mitochondrial function in circulating cells and that in the heart remains poorly defined and is a major obstacle to developing a reliable blood-based diagnostic (122).
3. Pre-analytical and Analytical Standardization: There is a profound lack of standardization across the entire diagnostic workflow.

Pre-analytical variables: Sample collection, processing, storage, and isolation protocols (e.g., for PBMCs or mtDNA) vary widely between laboratories, significantly impacting the stability and measurement of biomarkers like mtDNA or proteins (120).

Analytical variability: For functional assays like Seahorse XF, there are no standardized protocols for cell numbers, plating densities, assay conditions, or data normalization. This leads to high inter-laboratory variability and prevents the establishment of robust reference ranges (119).

Heteroplasmy quantification: Detecting and quantifying low-level mtDNA heteroplasmy requires highly sensitive and specific techniques (e.g., digital droplet PCR, deep sequencing), which are not yet standardized or cost-effective for routine clinical use (123).

4. Absence of Well-Designed Prospective Clinical Trials: Most biomarker studies to date have been retrospective or cross-sectional (22). There is a critical need for large-scale, prospective, multi-center clinical trials designed to:

Validate the independent prognostic value of candidate mitochondrial biomarkers over and above established ones (e.g., troponin, NT-proBNP).

Determine specific cut-off values that correlate with hard clinical endpoints (e.g., infarct size, MACE, progression to heart failure).

Demonstrate that biomarker-guided interventions lead to improved patient outcomes.

#### 5.5.2 A pathway to regulatory approval: the need for multi-modal integration

Given the multifaceted nature of mitochondrial dysfunction, a single biomarker is unlikely to ever capture its full complexity. A more promising pathway to regulatory approval lies in the development of integrated diagnostic algorithms that combine information from multiple domains. This approach aligns with the FDA's and other regulators' growing interest in multi-modal and composite biomarkers (124).

A proposed framework for regulatory approval involves the following steps:

1. Development of a Multi-Omic Panel: The first step is to move beyond single biomarkers and develop a validated panel of circulating factors that reflect different facets of mitochondrial pathology. This panel could include:

Mitochondrial DNA markers: Quantification of mtDNA-CN and detection of specific heteroplasmy levels (e.g., in *MT-TL1, MT-ND5*) in circulating leukocytes or plasma.

Protein biomarkers: A combination of mitochondrial-derived proteins, such as ATP5B (reflecting complex V integrity), GDF-15 (a stress-responsive mitokine), and S100A8/A9 (reflecting inflammation-induced mitochondrial dysfunction).

Metabolomic signatures: Identification of a specific metabolic fingerprint indicative of impaired oxidative phosphorylation, such as an elevated lactate/pyruvate ratio, altered ketone bodies, or specific acylcarnitine profiles (125).

2. Integration with Imaging Biomarkers: A blood-based panel alone may be insufficient. Its diagnostic and prognostic power would be significantly enhanced by integrating it with advanced, non-invasive imaging techniques that provide spatial and functional information about the heart.

Cardiac MRI: Parameters like infarct size, microvascular obstruction, and extracellular volume provide a structural and tissue-level correlate of mitochondrial injury (126).

Novel PET Tracers: The incorporation of novel PET tracers specifically targeting mitochondrial function, such as 18F-BMS-747158-02 (which targets mitochondrial complex I), could offer a direct, non-invasive readout of myocardial mitochondrial bioenergetics. The combination of a sensitive blood-based panel with a specific functional imaging readout would create a powerful diagnostic synergy.

3. Establishing a “Mitochondrial Dysfunction Score”: Using advanced statistical methods like machine learning, the data from the multi-omic panel and the imaging parameters would be combined to derive a composite “Mitochondrial Dysfunction Score.” This score would be designed to:

Provide a probability of significant myocardial mitochondrial involvement in the acute MI setting.

Stratify patients into risk categories for adverse post-MI outcomes (e.g., heart failure, malignant arrhythmias).

Serve as a companion diagnostic to identify patients most likely to respond to specific mitochondria-targeted therapies (e.g., those with a high “oxidative stress” sub-score might benefit from an antioxidant like MitoQ, while those with a low “biogenesis” sub-score might be candidates for PGC-1α enhancers) (127).

4. Validation in Prospective Registries and Trials: The final and most critical step is to validate this composite score in large, prospective, multi-center studies. This would involve:

Analytical validation: Ensuring the reproducibility and robustness of each component assay across different sites.

Clinical validation: Demonstrating that the score independently predicts clinical outcomes and improves risk reclassification compared to current standards.

Clinical utility: Ultimately, a randomized controlled trial would be needed to show that using this score to guide therapeutic decisions (e.g., selection for a clinical trial of a mitochondrial drug) leads to better patient outcomes than standard care (128).

By embracing this integrated, multi-modal approach—combining the “what” (circulating biomarkers), the “where” (imaging), and the “how” (functional assays in development)—the field can overcome the limitations of single biomarkers and pave a viable path toward regulatory approval and the introduction of clinically meaningful diagnostic tools for mitochondrial dysfunction in MI.

## 6 Therapeutic strategies targeting mitochondrial dysfunction in MI

### 6.1 Pharmacological interventions for mitochondrial dysfunction in MI

Pharmacological interventions aimed at addressing mitochondrial dysfunction in MI have demonstrated potential in numerous studies. Anesthetics such as sevoflurane and propofol have been explored for their cardioprotective properties (129, 130). In a comparative study utilizing a rabbit model of myocardial I/R injury, it was observed that sevoflurane anesthesia significantly reduced myocardial infarct size relative to propofol anesthesia. Mitochondria from animals subjected to propofol anesthesia exhibited a markedly decreased mitochondrial respiratory control ratio and impaired activities of respiratory complexes I and IV. In contrast, such mitochondrial dysfunction was not observed in animals anesthetized with sevoflurane (131).

Statins, which are extensively utilized for the reduction of low-density lipoprotein levels and the prevention of MI and cerebrovascular accidents, have also been investigated concerning mitochondrial dysfunction (132). Despite concerns regarding the heightened risk of diabetes mellitus associated with prolonged statin therapy, evidence suggests that atorvastatin confers cardioprotection against I/R-induced injury by inhibiting the opening of the mPTP through the activation of mitochondrial ATP-sensitive potassium (mitoKATP) channels. This finding implies that statins may exert a favorable impact on mitochondrial function in the context of MI (25).

However, the mitochondrial effects of statins are not exclusively beneficial. Statin-associated myopathy, a common adverse effect, has been linked to mitochondrial dysfunction through multiple mechanisms. Statins inhibit the synthesis of coenzyme Q10 (CoQ), an essential electron carrier in the mitochondrial electron transport chain (ETC), leading to impaired oxidative phosphorylation and reduced ATP production in skeletal muscle (132). Additionally, statins may directly compromise ETC complex activity, increase mitochondrial ROS production, and induce mitochondrial membrane depolarization, thereby promoting myocyte apoptosis and necrosis (133, 134). These mitochondrial perturbations are thought to underlie symptoms ranging from mild myalgia to life-threatening rhabdomyolysis, particularly in susceptible individuals.

Balancing the cardiovascular benefits of statins with their potential mitochondrial toxicity remains a clinical challenge. The cardioprotective effects of statins, including plaque stabilization, anti-inflammatory actions, and endothelial function improvement, often outweigh the risks of myopathy in high-risk cardiovascular patients. However, in patients presenting with unexplained muscle symptoms, alternative strategies should be considered. These include switching to a different statin (e.g., pravastatin or rosuvastatin, which have lower mitochondrial affinity), reducing the dose, or using intermittent dosing regimens (135). Supplementation with CoQ has been explored as a potential mitigator of statin-induced mitochondrial dysfunction, though clinical evidence remains inconclusive (136).

Monitoring strategies for statin-associated myopathy should include regular assessment of muscle symptoms, particularly in patients with risk factors such as advanced age, renal impairment, hypothyroidism, or concomitant use of drugs that interfere with statin metabolism (e.g., fibrates, cyclosporine). Measurement of creatine kinase levels is recommended in symptomatic patients to confirm muscle injury and guide treatment adjustments (137). Early recognition and individualized management of statin-induced mitochondrial myopathy are essential to maintain adherence to lipid-lowering therapy while minimizing adverse effects.

In addition, agents targeting oxidative stress, such as antioxidants, have been explored, given that oxidative stress is a significant contributor to mitochondrial dysfunction in MI (133, 134). For instance, melatonin has been demonstrated to activate the SIRT1-PGC-1α-SIRT3 signaling pathways following isoproterenol-induced myocardial injury in rat models, thereby mitigating oxidative stress, enhancing superoxide dismutase activity, and preserving mitochondrial architecture and function (135).

Moreover, pharmacological agents that influence mitochondrial dynamics are currently under investigation. Specifically, mitochondrial fission inhibitors, such as Mdivi-1, and mitochondrial fusion promoters, such as M1, have demonstrated efficacy in enhancing cardiac mitochondrial function, reducing infarct size, and preserving left ventricular function in animal models of cardiac I/R injury (136, 137).

### 6.2 Gene therapy approaches for mitochondrial dysfunction in MI

Gene therapy strategies aimed at addressing mitochondrial dysfunction in the context of MI are gaining recognition as promising therapeutic avenues. A key area of investigation involves the manipulation of genes associated with mitochondrial function (78, 102, 138). Notably, the cardiac-specific overexpression of NADH:ubiquinone oxidoreductase core subunit S1 (Ndufs1) has demonstrated efficacy in mitigating cardiac dysfunction and myocardial fibrosis during the reparative phase following MI. The overexpression of Ndufs1 has been shown to reduce MI/hypoxia-induced ROS production and ROS-related apoptosis, while also enhancing the diminished activity of complex I and ameliorating impaired mitochondrial respiratory function (138).

An alternative strategy involves targeting genes associated with mitochondrial biogenesis. The peroxisome proliferator-activated receptor gamma coactivator 1α (PGC-1α) serves as a pivotal regulator of mitochondrial biogenesis (139, 140). Nevertheless, efforts to enhance PGC-1α expression as a therapeutic intervention have yielded inconsistent results. While moderate cardiac-specific overexpression of PGC-1α improved mitochondrial and cardiac function in young mice, it expedited cardiac aging and reduced lifespan in older mice. This underscores the necessity for precise modulation of gene expression in a context-dependent manner to achieve effective therapeutic outcomes (141).

Furthermore, gene therapy aimed at targeting mtDNA mutations presents a promising avenue for research. Mutations in mtDNA have been linked to mitochondrial dysfunction and an elevated risk of MI (142). Despite the existing challenges in effectively delivering therapeutic genes to mitochondria, progress in gene delivery technologies may eventually surmount these barriers. A comprehensive understanding of the role of mtDNA mutations in MI, along with the development of strategies to correct or mitigate these mutations, could potentially result in innovative therapeutic approaches (143).

### 6.3 Lifestyle modifications and mitochondrial health in MI

Lifestyle modifications are integral to preserving mitochondrial health and may significantly mitigate the risk of MI (144, 145). Dietary habits constitute a critical component of these lifestyle changes. Research examining the relationship between dietary quality before and after MI and its impact on all-cause and cardiovascular mortality among MI survivors indicates that individuals adhering to a higher-quality diet, as assessed by the Alternative Healthy Eating Index 2010, exhibit reduced subsequent all-cause mortality. A diet of superior quality may enhance mitochondrial function by supplying essential nutrients and antioxidants, thereby diminishing oxidative stress and supporting mitochondrial metabolism (146).

Physical exercise represents a crucial lifestyle modification. Consistent physical activity has been shown to enhance mitochondrial homeostasis within the cardiac tissue (144). Specifically, aerobic interval training (AIT) has demonstrated efficacy in mitigating mitochondrial dysfunction in rats following MI. AIT achieves restoration of post-MI mitochondrial function by inhibiting pathological remodeling of mitochondrial dynamics, which correlates with the inactivation of the ERK1/2-JNK-P53 signaling pathway and an upregulation of nuclear PGC-1α expression (147). Additionally, smoking cessation is of paramount importance. Smoking constitutes a significant risk factor for MI and is linked to elevated oxidative stress and mitochondrial dysfunction (142, 148). Discontinuing smoking can decrease oxidative stress, enhance endothelial function, and potentially restore mitochondrial function (149, 150).

Furthermore, the management of stress may exert a positive influence on health outcomes. Chronic stress has been linked to unfavorable long-term consequences following AMI. Elevated levels of perceived stress during an AMI have been correlated with increased mortality rates over a two-year period and diminished health status after one year (151, 152). Implementing stress management strategies, including relaxation techniques and psychological counseling, may mitigate the detrimental effects on mitochondrial function and enhance the overall prognosis (151).

### 6.4 Clinical trials of mitochondrial-targeted therapies and mitochondrial transplantation

#### 6.4.1 Summary and analysis of major clinical trials

Despite promising preclinical results, translating mitochondrial-targeted therapies into clinical success for MI has proven challenging. Several key trials highlight both the potential and the hurdles.

Elamipretide (SS-31): This mitochondria-targeting peptide antioxidant showed significant cardioprotection in animal models of I/R injury (153, 154). However, clinical trials in patients with AMI or heart failure have yielded mixed or neutral results regarding primary endpoints like left ventricular ejection fraction improvement or clinical outcomes (155–157). Potential reasons for this disconnect include: significant patient heterogeneity (comorbidities, age) not reflected in animal models, suboptimal timing of administration relative to the ischemic insult in a clinical setting, and differences in pharmacokinetics and mitochondrial bioavailability between species.

MitoQ (Mitoquinone mesylate): As a mitochondria-targeted antioxidant (Coenzyme Q10 analog), MitoQ demonstrated benefits in endothelial function and reduced biomarkers of oxidative stress in early-phase studies (158). However, larger trials in conditions like Parkinson's disease or in cardiovascular prevention have not shown definitive clinical efficacy for hard cardiac endpoints post-MI. Limitations may involve inadequate targeting efficiency to ischemic cardiomyocytes in humans, variable baseline mitochondrial function among participants, and the multifactorial nature of post-MI remodeling where single-target antioxidant therapy may be insufficient.

Drugs Modulating Mitochondrial Dynamics: Pharmacological agents like the fission inhibitor Mdivi-1 have shown robust efficacy in reducing infarct size and improving heart function in animal models (159, 160). However, they have not yet advanced to late-stage clinical trials for MI, primarily due to challenges in achieving cardiac-specific delivery, potential off-target effects, and a lack of validated biomarkers to identify patients who would benefit most from such modulation.

The translational challenges associated with mitochondrial-targeted therapies primarily arise from several factors (1): the discrepancies between controlled animal models and the clinically complex, comorbid patient populations (2); the critical yet frequently overlooked therapeutic window during early reperfusion in clinical settings (3); the absence of precise, non-invasive diagnostic tools for stratifying patients according to the degree or specific type of mitochondrial dysfunction; and (4) the inefficient and non-specific delivery of therapeutics to cardiac mitochondria.

#### 6.4.2 Mitochondrial transplantation: an emerging therapeutic avenue

Mitochondrial transplantation represents a novel, direct strategy to replenish dysfunctional mitochondria with healthy exogenous ones, aiming to restore bioenergetics and cellular viability.

Mechanism and Preclinical Evidence: Studies demonstrate that isolated autologous or allogeneic mitochondria can be internalized by cardiomyocytes when delivered directly into the ischemic zone (161). This internalization leads to improved ATP production, reduced ROS, suppression of apoptosis, and enhanced cell survival (162). In animal models of I/R injury, intramyocardial injection of mitochondria significantly improved cardiac function and reduced infarct size (163).

Early Clinical Exploration: Preliminary clinical applications have emerged primarily in pediatric cardiac surgery for congenital heart disease, where autologous mitochondrial transplantation into the myocardium appeared safe and was associated with improved metabolic parameters and ventricular function (163, 164). For adult AMI, clinical experience remains very limited but is the focus of growing investigative interest, particularly in the context of refractory cardiogenic shock or during cardiac surgery (164, 165).

Challenges and Future Directions:

Source and Immunology: While autologous transplantation avoids immune rejection, the patient's own mitochondria may be inherently compromised. Allogeneic sources raise questions of immunogenicity and long-term compatibility.

Delivery and Retention: Current methods (direct intramyocardial injection, intracoronary infusion) are invasive or may have low retention rates. Developing efficient, minimally invasive, and targeted delivery systems is crucial.

Standardization and Scaling: Protocols for mitochondrial isolation, quality assessment, dosage, and transplantation need standardization for clinical reproducibility.

Integration and Mechanism: The long-term fate, functional integration, and persistence of transplanted mitochondria within host cells require further elucidation.

Future strategies may involve combining mitochondrial transplantation with biomaterial scaffolds (e.g., hydrogels for sustained release), cell-based carriers, or nanotechnology to enhance targeting and retention. Rigorously designed controlled clinical trials are essential to definitively establish the safety, efficacy, and optimal application protocols of mitochondrial transplantation for MI patients (Table 3).

**Table 3:** Therapeutic strategies targeting mitochondrial dysfunction in myocardial infarction.

Strategy/agent	Mechanism of action	Trial phase/evidence level	Key outcomes/efficacy	Major limitations/challenges
Elamipretide (SS-31)	Cardiolipin-binding peptide; stabilizes mitochondrial cristae, reduces ROS	Phase 1/2 in AMI and HF	Cardioprotection in animal models; neutral results in clinical trials	Patient heterogeneity, suboptimal dosing timing, species differences
MitoQ	Mitochondria-targeted antioxidant (CoQ10 analog)	Phase 2 in cardiovascular and metabolic diseases	Improved endothelial function; no definitive post-MI hard endpoint benefit	Targeting efficiency, multifactorial post-MI pathology
Mdivi-1	Inhibits mitochondrial fission (Drp1)	Preclinical	Reduces infarct size, preserves LV function in I/R models	Lack of cardiac-specific delivery; no late-stage trials
Statins (e.g., atorvastatin)	Activate mitoKATP channels; inhibit mPTP opening; also impair CoQ10 synthesis	Established clinical use	Cardioprotective in I/R; reduces MI risk	Dual effect: benefits vs. mitochondrial myopathy; requires monitoring
Mitochondrial Transplantation	Direct transfer of healthy mitochondria into ischemic myocardium	Early clinical (pediatric cardiac surgery); preclinical for adult MI	Restores ATP, reduces apoptosis, improves function in small studies	Delivery method, retention, immune response, scalability
Gene Therapy (e.g., Ndufs1, PGC-1α)	Overexpression of mitochondrial genes to enhance biogenesis or ETC function	Preclinical	Improved mitochondrial respiration, reduced apoptosis in animal models	Delivery safety, off-target effects, context-dependent outcomes
Lifestyle (exercise, diet)	AMPK-PGC-1α pathway activation; enhances biogenesis and mitophagy	Observational/supportive	Improved mitochondrial homeostasis, reduced mortality post-MI	Requires long-term adherence; not a standalone acute therapy

## 7 Mitochondrial biogenesis, mitophagy, and clearance pathways: mechanisms and therapeutic candidates

Mitochondrial homeostasis is preserved through a finely tuned equilibrium between biogenesis, which refers to the production of new mitochondria, mitophagy, the selective autophagic removal of damaged mitochondria, and various other clearance mechanisms (113). The disruption of these processes is a characteristic feature of mitochondrial dysfunction in MI (78). This section elucidates the principal pathways involved and evaluates potential therapeutic candidates designed to augment these protective mechanisms.

### 7.1 Mitochondrial biogenesis pathways and enhancing strategies

Mitochondrial biogenesis is predominantly governed by the PGC-1α signaling pathway (166). In response to increased energy demands or stress stimuli, such as physical exercise or caloric restriction, PGC-1α co-activates nuclear respiratory factors 1 and 2 (NRF1 and NRF2). This activation subsequently enhances the expression of nuclear-encoded mitochondrial proteins and the mitochondrial transcription factor A (TFAM), which is crucial for the replication and transcription of mtDNA (167, 168). Critical upstream regulators of this process include AMP-activated protein kinase (AMPK), which activates PGC-1α under conditions of low energy availability, and sirtuin 1 (SIRT1), which deacetylates and activates PGC-1α in response to fluctuations in NAD + levels (169, 170).

Therapeutic Candidates to Enhance Biogenesis:

Pharmacological Activators: Compounds including resveratrol, a SIRT1 activator, and AICAR, an AMPK activator, have demonstrated potential in preclinical models by upregulating PGC-1α and enhancing mitochondrial function following MI (171, 172). Although primarily recognized for its role in inhibiting mitochondrial fission, the mitochondrial division inhibitor Mdivi-1 has also been associated with enhanced mitochondrial biogenesis in certain contexts, attributed to its ability to reduce the clearance of healthy mitochondria (173).

Gene Therapy: In animal models of heart failure, cardiac-specific overexpression of PGC-1α or its upstream regulators, such as AMPK, has been shown to enhance mitochondrial content and function (174). However, careful consideration of dosage and timing is essential to prevent potential adverse effects, including accelerated cardiac aging.

Lifestyle Interventions: Aerobic exercise remains one of the most potent physiological stimulators of mitochondrial biogenesis via the AMPK-PGC-1α signaling pathway (175).

### 7.2 Mitophagy pathways and pharmacological modulation

Mitophagy, a selective form of autophagy, is crucial for removing damaged mitochondria to prevent the accumulation of dysfunctional organelles and the release of pro-death factors. Two primary pathways are implicated in MI:

PINK1/Parkin Pathway: Under conditions of mitochondrial depolarization, the PTEN-induced kinase 1 (PINK1) becomes stabilized on the outer mitochondrial membrane, where it facilitates the recruitment of the E3 ubiquitin ligase Parkin. Subsequently, Parkin ubiquitinates proteins located on the outer membrane, thereby tagging the mitochondrion for degradation through autophagy (176, 177). This process involves the engagement of LC3-binding adaptor proteins, such as p62, which mediate the engulfment of the marked mitochondrion by autophagosomes.

Receptor-Mediated Pathway: Mitophagy receptors, including BCL2/adenovirus E1B 19 kDa protein-interacting protein 3 (BNIP3), NIP3-like protein X (NIX/BNIP3L), and FUN14 domain-containing 1 (FUNDC1), are capable of directly interacting with LC3 on autophagosomal membranes independently of Parkin, particularly in response to hypoxic conditions or cellular stress (178, 179).

Therapeutic Candidates to Enhance Mitophagy:

Small Molecule Inducers: Urolithin A, a natural metabolite, has been shown to induce mitophagy and improve mitochondrial function in aged animals and models of muscular degeneration; its potential in post-MI cardiac repair is under investigation (180, 181). The compound spermidine, a natural polyamine, also promotes autophagy and mitophagy, extending lifespan and improving cardiac function in models of aging and pressure overload (182, 183).

Gene and Peptide-Based Approaches: Overexpression of PINK1, Parkin, or FUNDC1 has been protective in I/R injury models by enhancing the clearance of damaged mitochondria (184, 185). Additionally, peptides mimicking the LC3-interacting region (LIR) of mitophagy receptors are being explored as targeted inducers (186).

Indirect Modulators: Pharmacological agents that enhance autophagic flux, exemplified by rapamycin (an mTOR inhibitor), have been shown to augment mitophagy as a component of a comprehensive cellular clearance mechanism, demonstrating advantageous effects in preclinical models of cardiac aging and MI (187, 188).

### 7.3 Mitochondrial clearance and quality control: beyond mitophagy

While mitophagy is the primary clearance route for whole damaged mitochondria, other quality control mechanisms operate at the sub-organellar level:

Mitochondrial-Derived Vesicles (MDVs): These vesicles bud off from mitochondria to deliver oxidized proteins or specific components to lysosomes or peroxisomes for degradation, acting as a “piecemeal” cleanup system before whole-organelle autophagy is triggered (189).

The Mitochondrial Unfolded Protein Response (mtUPR): This stress response pathway, activated by the accumulation of unfolded proteins within mitochondria, upregulates nuclear-encoded mitochondrial chaperones and proteases to restore protein homeostasis (190). Enhancing mtUPR capacity is a novel therapeutic concept for diseases involving mitochondrial proteotoxic stress.

Proteolytic Systems: Lon and AAA proteases within the mitochondrial matrix degrade misfolded or damaged proteins. Their activity can be compromised in MI, and strategies to support their function are of interest (191, 192).

Therapeutic Implications:

The exploration of auxiliary clearance pathways represents a novel frontier in research. Compounds that activate the mtUPR, such as the NAD + precursor nicotinamide riboside, or those that enhance mitochondrial protease function, may offer complementary advantages in conjunction with mitophagy inducers, thereby establishing a multi-faceted defense mechanism against mitochondrial damage following MI. The development of biomarkers specific to these pathways, such as circulating MDV profiles and mtUPR gene signatures, will be essential for patient stratification and for monitoring therapeutic efficacy.

## 8 Controversies and challenges in mitochondrial dysfunction research in MI

### 8.1 Debates on the causative role of mitochondrial dysfunction in MI

The causative role of mitochondrial dysfunction in MI remains a topic of debate. Several studies have provided compelling evidence suggesting that mitochondrial dysfunction is a critical contributor to MI (193). For instance, research investigating the role of Scpep1 in MI demonstrated that Scpep1 exacerbates MI-induced cardiac dysfunction and damage by impairing mitochondrial bioenergetics, which subsequently leads to mitochondrial fission and apoptosis. Notably, the genetic ablation or cardiac-specific knockdown of Scpep1 mitigated these detrimental effects, thereby indicating a causal relationship between mitochondrial dysfunction and MI (106).

Conversely, some scholars propose that mitochondrial dysfunction may be more accurately characterized as a consequence rather than a primary cause (20, 62). In certain instances, alternative factors such as oxidative stress, inflammation, and calcium overload may initiate the pathological process of MI, with mitochondrial dysfunction emerging as a secondary phenomenon (63, 64). For example, in the context of sepsis-induced myocardial dysfunction, mitochondrial dysfunction is implicated; however, it occurs within a complex interplay of factors, including dysregulated immune responses, metabolic reprogramming, and the excessive production of ROS (65).

Another dimension of the discourse pertains to the precise mechanisms through which mitochondrial dysfunction contributes to MI. Numerous studies have delineated pathways including mitochondrial ROS production, mPTP opening, and imbalances in mitochondrial dynamics. However, the definitive sequence and relative significance of these mechanisms remain inadequately elucidated. For instance, although mitochondrial ROS production is frequently linked to MI, it remains uncertain whether it serves as the primary catalyst or as a downstream effect of other initial insults (67).

### 8.2 Challenges in translating mitochondrial research to clinical practice in MI

The translation of mitochondrial research into clinical practice for MI encounters several significant challenges. A primary challenge lies in the intricate nature of mitochondrial function and dysfunction. Mitochondria perform a multitude of functions, and their dysfunction can arise from various factors, including mutations in mtDNA and nuclear DNA, as well as responses to aging and disease-related stresses (113). Developing mitochondria-targeted therapeutic strategies that effectively address this complexity is challenging, as diverse etiologies may necessitate distinct approaches (114).

Another critical challenge is the issue of drug delivery. The precise delivery of therapeutic agents to cardiac tissues and damaged mitochondria is essential for achieving successful clinical outcomes. However, existing techniques often lack the precision required for efficient and targeted delivery. For instance, in the context of gene therapy, the safe and effective delivery of genes to mitochondria remains a substantial obstacle (17).

The translation of preclinical findings into clinical applications presents significant challenges. Numerous treatments that demonstrate substantial cardioprotective effects in experimental animal models of acute ischemia and reperfusion injury have not replicated these benefits in clinical trials involving patients with AMI (68, 70). This discrepancy may be attributed to inherent differences between animal models and human patients, including variations in genetic background, the presence of comorbidities, and the complexity of the human cardiovascular system (71).

Furthermore, the absence of standardized diagnostic and prognostic tools for assessing mitochondrial dysfunction in the context of MI further impedes translational efforts. The development and implementation of effective therapeutic strategies are hampered by the lack of accurate and reliable methods for diagnosing mitochondrial dysfunction and predicting its progression in patients (22, 114).

Beyond these technical and biological hurdles, the clinical translation of emerging mitochondrial therapies—particularly gene-editing approaches—is further constrained by a highly divergent and restrictive global regulatory landscape. Despite the therapeutic promise of technologies such as CRISPR-Cas for correcting pathogenic mtDNA mutations, their clinical application, especially involving the germline, is governed by stringent and region-specific frameworks. In Europe, the regulatory landscape is notably prohibitive. The European Union's pharmaceutical legislation classifies products containing or consisting of genetically modified organisms (GMOs) as advanced therapy medicinal products (ATMPs), requiring centralized authorization. More fundamentally, the Oviedo Convention of the Council of Europe explicitly prohibits interventions on the human germline, effectively rendering any clinical application of heritable mitochondrial gene editing impermissible (194). In contrast, other regions have adopted different postures. The United Kingdom, while a signatory to the Oviedo Convention, has uniquely legalized mitochondrial replacement therapy (MRT) under the Human Fertilization and Embryology Act 1990, allowing it in strictly regulated, licensed clinics to prevent the transmission of serious mitochondrial disease, though this constitutes mitochondrial replacement rather than gene editing *per se*. In the United States, a report from the National Academies of Sciences, Engineering, and Medicine cautiously stated that clinical trials for MRT could be ethically permitted under strict oversight, but a legislative moratorium effectively prevents the Food and Drug Administration from considering such applications (195). Meanwhile, other countries, including Japan and Australia, are developing their own regulatory pathways for MRT, while China has reported controversial instances of gene-editing experiments, underscoring a fragmented and rapidly evolving international landscape. It is crucial to clarify that, regardless of jurisdiction, all clinical applications of mitochondrial gene editing or replacement remain experimental, are subject to rigorous oversight, and are permitted only under exceptional, highly regulated circumstances, with heritable modifications facing the greatest restrictions. Navigating this complex regulatory and ethical terrain is as critical as overcoming the technical and biological barriers for the future of mitochondrial medicine in MI.

## 9 Limitations and future directions in mitochondrial dysfunction and MI

### 9.1 Emerging technologies in mitochondrial dysfunction research for MI

Emerging technologies are creating new opportunities in the study of mitochondrial dysfunction related to MI. One notable advancement is the development of ROS-responsive liposomal composite hydrogels (196, 197). A recent study has reported the synthesis of a ROS-responsive PAMB-G-TK/4-arm-PEG-SG hydrogel designed for the localized delivery of drug-loaded liposomes. These liposomes encapsulate elamipretide (SS-31) to mitigate mitochondrial oxidative damage and sphingosine-1-phosphate (S1P) to stimulate angiogenesis. This hydrogel has shown efficacy in targeting the mitochondria of damaged cardiomyocytes, ameliorating mitochondrial dysfunction, and promoting angiogenesis in a rat model of MI (196).

Another promising area of research involves the application of neutrophil-derived apoptotic body membrane-fused exosomes. The fusion of exosomes with neutrophil-derived apoptotic body membranes enhances their targeting specificity and retention within infarcted myocardium (198, 199). In a mouse model of MI, these NAM-fused exosomes (NAM-Exo) not only modulated inflammatory responses but also facilitated angiogenesis, resulting in improved cardiac function and ventricular remodeling (198).

Recent advancements in molecular imaging techniques have significantly enhanced our understanding of underlying biological processes. Specifically, the integration of targeted imaging agents with modalities such as MRI, single-photon emission computed tomography (SPECT), and PET facilitates the visualization of molecular processes involved in post-MI immune cell response and tissue remodeling. This approach contributes to elucidating the role of mitochondrial dysfunction within the broader pathological framework (109).

Furthermore, the advent of novel gene-editing technologies, notably CRISPR-Cas systems, presents the potential to rectify mtDNA mutations linked to MI (200, 201). Despite the persisting technical and ethical challenges, these technologies offer promising avenues for future therapeutic interventions.

However, the clinical translation of these emerging technologies will require rigorous standardization of manufacturing protocols, quality control metrics, and delivery systems to ensure reproducibility across different centers and patient populations.

### 9.2 Potential for personalized medicine approaches in mitochondrial dysfunction and MI

Personalized medicine strategies in addressing mitochondrial dysfunction and MI hold significant promise for transforming therapeutic approaches. A key component involves utilizing genetic information to customize treatments. Given that mutations and variations in mtDNA have been linked to an elevated risk of MI, a comprehensive understanding of an individual's genetic profile could facilitate the development of targeted therapeutic strategies (202, 203). For instance, in cases where a patient possesses a specific mtDNA mutation, gene-based therapies may be devised to rectify or mitigate the effects of this mutation (204).

Additionally, the application of biomarkers to inform personalized treatment is another critical area of focus. Biomarkers such as ATP5B, S100a8/a9, and genes related to mitochondrial function can aid in predicting MI risk, disease progression, and treatment response (11, 205). By evaluating a patient's biomarker profile, clinicians can tailor treatment plans, selecting the most suitable pharmacological agents or lifestyle interventions (12, 97).

Moreover, personalized medicine may encompass the consideration of an individual's lifestyle, comorbidities, and environmental factors (206). For instance, patients engaged in high-risk behaviors, such as smoking, obesity, and physical inactivity, might benefit from more intensive lifestyle modification programs. Conversely, individuals with comorbid conditions like diabetes or hypertension may necessitate a combination of therapeutic approaches that concurrently address both the comorbidity and mitochondrial dysfunction.

Additionally, the burgeoning field of metabolomics holds potential contributions to personalized medicine. Metabolomics can elucidate dynamic metabolic alterations associated with diseases, thereby providing insights into tailored treatment strategies (207). By examining an individual's metabolite profile, it may be feasible to identify specific metabolic pathways disrupted in conditions such as mitochondrial dysfunction and MI, facilitating the development of targeted therapies (208).

Crucially, the successful implementation of personalized medicine depends on establishing clinically actionable thresholds for mitochondrial biomarkers. Future studies must define the specific cut-off values for mtDNA-CN, heteroplasmy levels, or protein biomarkers that warrant intervention or predict treatment response. This will require large-scale, multi-center cohorts to generate robust reference ranges and validate their utility across diverse populations.

### 9.3 Long—term prognosis and mitochondrial health in MI survivors

The long-term prognosis of individuals who have survived a MI is intricately linked to the health of their mitochondria. Research indicates that variables such as dietary habits, physical activity, and stress management significantly influence mitochondrial function, thereby affecting long-term outcomes (151, 209). For instance, MI survivors adhering to a higher-quality diet exhibit reduced all-cause mortality rates, implying that dietary factors may enhance mitochondrial health and subsequently improve prognosis (146).

Physical exercise is also a pivotal factor; regular engagement in exercise has been shown to enhance mitochondrial homeostasis, which may, in turn, lead to improved long-term cardiac function (210). Specifically, aerobic interval training has demonstrated efficacy in mitigating mitochondrial dysfunction in post-MI rat models, suggesting potential benefits for long-term prognosis (147).

Conversely, factors that impair mitochondrial function, such as chronic stress, can adversely affect the long-term prognosis (211). Elevated levels of perceived stress during an AMI are linked to unfavorable long-term outcomes, including increased mortality and diminished health status (151).

Furthermore, the presence of comorbidities, such as diabetes and heart failure, can also significantly influence the long-term prognosis of MI survivors (212). For instance, diabetes is associated with a heightened risk of mortality following MI, a risk that may be influenced by left ventricular function. A comprehensive understanding of the intricate interactions between mitochondrial dysfunction, comorbidities, and long-term prognosis is crucial for the development of effective treatment and management strategies for MI survivors (213).

To translate these observations into clinical practice, future longitudinal studies must employ standardized protocols for assessing mitochondrial function and incorporate repeated biomarker measurements to track dynamic changes over time. Multi-center registries with harmonized data collection are essential to identify the determinants of favorable vs. adverse mitochondrial remodeling post-MI.

### 9.4 Post-myocardial infarction cognitive decline: the heart-brain axis and mitochondrial link

Emerging evidence suggests that the detrimental effects of mitochondrial dysfunction and systemic inflammation following MI extend beyond the heart to the brain, contributing to neurocognitive decline and mild cognitive impairment (MCI) in survivors. This heart-brain axis represents an underexplored but clinically significant dimension of post-MI recovery (214).

#### 9.4.1 Mechanistic links: systemic inflammation, mitochondrial dysfunction, and cognitive decline

The pathophysiology of post-MI cognitive impairment is multifactorial, involving a synergistic interplay between systemic inflammation and mitochondrial dysfunction:

1. Systemic Inflammatory Cascade: Acute MI triggers a robust systemic inflammatory response characterized by the release of pro-inflammatory cytokines (e.g., IL-1β, IL-6, TNF-α) and damage-associated molecular patterns (DAMPs), including circulating cell-free mtDNA. This systemic inflammation can compromise the blood-brain barrier (BBB), allowing inflammatory mediators to infiltrate the central nervous system (CNS) and activate microglia, the resident immune cells of the brain. Proteins like S100A8/A9, identified earlier as a key player in MI, act as potent DAMPs in this context, linking cardiac injury to neuroinflammation (215, 216).
2. Mitochondrial Dysfunction in the Brain: Chronic neuroinflammation induces mitochondrial dysfunction in neuronal and glial cells (217). Activated microglia undergo metabolic reprogramming and produce excessive ROS, which can damage neuronal mtDNA, impair oxidative phosphorylation, and disrupt mitochondrial dynamics (fission/fusion) within the brain. This leads to neuronal energy deficit, synaptic dysfunction, and eventual neurodegeneration, underpinning cognitive deficits.
3. Vascular Cognitive Impairment: MI is often a manifestation of systemic atherosclerosis. Microvascular damage and endothelial dysfunction, exacerbated by mitochondrial oxidative stress, are common to both the heart and the brain. Cerebral small vessel disease, driven by these same mechanisms, can lead to white matter lesions and subcortical ischemic damage, further contributing to cognitive decline independent of recurrent stroke (214).

#### 9.4.2 Biomarker strategies for identifying at-risk patients

Early identification of MI survivors at risk for cognitive decline is crucial for implementing preventive strategies. A multi-modal biomarker approach is recommended:

1. Blood-Based Biomarkers:

Inflammatory Markers: Persistent elevation of high-sensitivity C-reactive protein (hs-CRP), IL-6, and the S100A8/A9 complex beyond the acute phase may indicate ongoing systemic inflammation that threatens cerebral health (215, 216). Pentraxin 3 (PTX3), a vascular-specific inflammatory biomarker, has also been identified as a potential tool for recognizing post-MI cognitive decline (214).

Mitochondrial Markers: Low mtDNA-CN in peripheral blood leukocytes, a surrogate for systemic mitochondrial dysfunction, has been associated not only with cardiovascular outcomes but also with cognitive impairment. Elevated levels of circulating cell-free mtDNA may serve as a DAMP that predicts neuroinflammation.

Neuronal Injury Markers: Plasma levels of neurofilament light chain (NfL), a marker of axonal injury, and glial fibrillary acidic protein (GFAP), a marker of astrocytic activation, have shown promise in detecting subclinical brain damage in cardiovascular disease cohorts (217).

2. Imaging Biomarkers:

Brain MRI: Routine brain MRI in high-risk MI survivors (e.g., those with heart failure, multiple comorbidities) can assess for silent brain infarcts, white matter hyperintensities (a marker of small vessel disease), and hippocampal atrophy, which are structural correlates of cognitive decline (214).

Advanced Imaging: Emerging PET tracers targeting mitochondrial function (e.g., 18F-BMS-747158-02 for complex I) or neuroinflammation (e.g., 18F-GE180 for TSPO) could potentially be adapted to assess the brain, providing a direct readout of cerebral mitochondrial health and microglial activation.

#### 9.4.3 Proposed follow-up protocols and preventive strategies

Integrating cognitive health into standard post-MI care pathways is essential.

1. Cognitive Screening: We propose a staged approach to cognitive assessment:

Baseline Screening: All MI survivors, particularly those aged >65 or with risk factors (history of stroke, heart failure, diabetes), should undergo baseline cognitive screening using brief, validated tools. Recent evidence suggests that attention is a primary cognitive domain affected post-MI, which can be assessed using specific components of the Mini-Mental State Examination (MMSE) alongside tools like the Clock Drawing Test (CDT) (218).

Targeted Neuropsychological Evaluation: Patients scoring below age-adjusted norms on screening or those reporting subjective cognitive complaints should be referred for comprehensive neuropsychological testing.

2. Longitudinal Monitoring:

High-risk patients identified by the biomarker panel (elevated NfL, persistent inflammation, low mtDNA-CN) or brain MRI findings should be monitored more closely with annual cognitive assessments.

Repeat assessment of blood-based biomarkers (e.g., NfL, inflammatory panel) during routine cardiovascular follow-ups (6–12 months) could provide dynamic information on ongoing neuroaxonal damage.

3. Therapeutic and Preventive Implications:

Lifestyle Interventions: The same lifestyle modifications that benefit mitochondrial health in the heart—aerobic exercise, Mediterranean diet, smoking cessation—are neuroprotective and should be strongly emphasized.

Pharmacological Strategies: While no specific therapies are approved for post-MI cognitive impairment, targeting the underlying mechanisms may offer benefit. For example, strategies aimed at reducing systemic inflammation (e.g., colchicine) or improving mitochondrial function (e.g., elamipretide) are currently under investigation in cardiovascular populations; their potential cognitive benefits should be explored as secondary outcomes in future trials.

Vascular Risk Factor Control: Rigorous management of hypertension, dyslipidemia, and diabetes remains the cornerstone for preventing both recurrent MI and vascular cognitive impairment (214).

By recognizing post-MI cognitive decline as a consequence of the systemic effects of mitochondrial dysfunction and inflammation, and by implementing systematic screening and biomarker-guided follow-up, clinicians can move towards a more holistic model of post-MI care that preserves both cardiac and brain health, ultimately improving the quality of life for survivors.

### 9.5 Addressing critical research gaps: standardization, validation, and clinical thresholds

Although the therapeutic targeting of mitochondrial dysfunction shows considerable promise, several critical gaps in current research need to be addressed to transform preclinical optimism into clinical success. These challenges will significantly shape the future direction of the field.

Towards the Development of Standardized Biomarker Panels: The current state of mitochondrial biomarkers, such as ATP5B, S100A8/A9, and mtDNA-CN, remains insufficient for clinical implementation due to the lack of standardized pre-analytical and analytical protocols. Variability in sample collection, processing, storage, and assay methods across studies precludes the establishment of universal reference ranges and thresholds (219, 220). Future efforts must prioritize the harmonization of these methodologies through consensus guidelines, inter-laboratory validation studies, and the development of certified reference materials. This includes standardizing protocols for mtDNA quantification, heteroplasmy detection, and protein biomarker measurement to ensure reproducibility and comparability across different centers and populations (221).

Confronting MI Heterogeneity through Multi-Center Validation: The significant heterogeneity observed in MI—encompassing aspects such as etiology, infarct territory, comorbid conditions (including diabetes and aging), and genetic background—substantially contributes to the variability in biomarker performance and therapeutic outcomes. Consequently, a universal approach to mitochondrial assessment or therapy is unlikely to be effective (219, 222). Large-scale, multi-center prospective cohorts are urgently needed to validate the diagnostic and prognostic utility of candidate biomarkers across diverse populations. Such studies must account for differences in age, sex, ethnicity, comorbidities, and medication use to ensure that findings are generalizable and that subgroup-specific effects are identified (220). Only through such rigorous validation can we move beyond single-center discoveries toward broadly applicable clinical tools.

Establishing Clinically Actionable Thresholds: A fundamental barrier to clinical implementation is the lack of clearly defined, clinically actionable thresholds for mitochondrial dysfunction. While reduced mtDNA-CN or elevated S100A8/A9 levels have been associated with adverse outcomes, the specific cut-off values that warrant intervention or predict treatment response remain undefined. Similarly, heteroplasmy levels that confer increased risk for MI or poor post-infarction recovery have not been systematically validated in large cohorts (223). Future research must focus on establishing evidence-based thresholds through well-designed prospective studies linking biomarker levels to hard clinical endpoints (e.g., infarct size, major adverse cardiovascular events, progression to heart failure, mortality) (224, 225). Advanced statistical methods, including machine learning and risk prediction modeling, should be employed to derive and validate these thresholds, ultimately enabling their incorporation into clinical decision-making algorithms (226).

Overcoming Translational Failures through Enriched Trial Design: The persistent inability to translate effective cardioprotective strategies from animal models to human clinical trials highlights a significant disconnect. To mitigate this issue, it is essential to develop preclinical models that more accurately reflect clinical conditions. This involves utilizing aged animal models, integrating comorbidities such as metabolic syndrome and hypertension, and employing chronic ischemia models that more closely replicate the progression of human disease. Additionally, advancements in targeted delivery systems, including ROS-responsive hydrogels and exosome-based carriers, should be prioritized to ensure adequate delivery of therapeutic agents to the compromised cardiomyocyte mitochondria in the challenging post-MI environment. Moreover, the design of clinical trials for mitochondrion-targeted therapies should incorporate enrichment strategies that utilize validated biomarkers and phenotypic stratification to identify patients most likely to respond, thereby increasing the likelihood of detecting a therapeutic effect in a responsive patient population.

Integrating Multi-Omic Approaches for Composite Signatures: Given the multifaceted nature of mitochondrial dysfunction, single biomarkers are unlikely to capture its full complexity. The future lies in the integration of multi-omics approaches—including proteomics, metabolomics, genomics, and epigenomics—with advanced analytical techniques, such as machine learning, to identify composite signatures. These signatures should not only facilitate the diagnosis of MI but also enable the stratification of its subtypes, predict the severity of mitochondrial injury, and monitor responses to mitochondrion-targeted therapies. By providing a dynamic, systems-level evaluation of mitochondrial health, such composite panels can transcend the limitations of single, nonspecific markers and pave the way for truly personalized risk stratification and therapeutic intervention.

Addressing these gaps—by developing standardized protocols, validating findings in multi-center cohorts, establishing clinically actionable thresholds, refining translational models, and embracing multi-omic integration—constitutes the most crucial frontier in advancing mitochondrial medicine from a compelling biological concept to a tangible clinical reality for patients with MI.

## 10 Conclusion

Mitochondrial dysfunction is unequivocally a pivotal factor in the pathogenesis and progression of MI. Nonetheless, the lack of standardized clinical tools for assessing mitochondrial health has resulted in a translational gap between preclinical discoveries and patient care. This review synthesizes a comprehensive range of evidence to identify which diagnostic markers and therapeutic strategies targeting mitochondria hold genuine promise and which encounter inherent limitations or have underperformed in translation. By elucidating the underlying reasons for these successes and failures—whether due to methodological challenges, patient heterogeneity, or biological complexity—we provide a rational framework for future research. Prioritizing the most robust biomarkers and translatable therapeutic mechanisms will be essential for designing informative clinical trials and advancing mitochondrial medicine toward meaningful clinical integration.

Therapeutic strategies targeting mitochondrial pathways—encompassing pharmacological agents, gene therapies, and lifestyle interventions—demonstrate considerable promise in preclinical models. Nonetheless, translating these findings into clinical practice remains challenging due to issues related to drug delivery, patient heterogeneity, and the complexity of mitochondrial biology. Additionally, ethical considerations in genetic and acute care research must be meticulously addressed to ensure the safe and equitable application of emerging therapies.

The prospective incorporation of emerging technologies, including mitochondrial-targeted nanotherapeutics, molecular imaging, and gene-editing tools, presents significant promise for the advancement of personalized medicine. Future efforts should aim to bridge the gap between preclinical research and clinical application, addressing current controversies and translational barriers. By utilizing individual genetic, metabolic, and lifestyle profiles, forthcoming interventions could be customized to enhance mitochondrial health and improve outcomes for MI survivors. Ongoing interdisciplinary research and collaborative initiatives are crucial to surmount current challenges and fully harness the therapeutic potential of addressing mitochondrial dysfunction in the context of MI.
